# Genomic Structural Equation Modeling Reveals Cardiovascular‐Kidney‐Metabolic Syndrome Genetic Architecture

**DOI:** 10.1111/1753-0407.70225

**Published:** 2026-04-13

**Authors:** Chuanlong Lu, Lizheng Li, Jinshan Chen, Runze Chang, Honglin Dong

**Affiliations:** ^1^ Department of Vascular Surgery, The Second Hospital Shanxi Medical University Taiyuan China

**Keywords:** cardiovascular‐kidney‐metabolic syndrome, genome‐wide association study, genomic structural equation modeling, single‐nucleotide polymorphisms, transcriptome‐wide association study

## Abstract

**Background:**

The genetic basis of cardiovascular‐kidney‐metabolic syndrome (CKMs) involves complex pleiotropy, necessitating analytical approaches capable of dissecting shared genetic architectures across multiple cardiometabolic traits.

**Methods:**

We employed genomic structural equation modeling (genomic SEM) to integrate summary statistics from six cardiometabolic traits. The model was assessed using standard fit indices. Genome‐wide association analyses were performed on 1,862,425 SNPs under stringent quality control measures, with LD Score regression applied to evaluate polygenic heritability and confounding bias. Novel loci were identified using GWAS‐by‐Subtraction. Functional characterization included transcriptome‐wide association analysis (TWAS), fine‐mapping, pathway enrichment analysis, and cell‐type specificity analysis.

**Results:**

The genomic SEM model demonstrated excellent fit (CFI = 0.99, SRMR = 0.14). Quality control metrics confirmed that genomic inflation (Lambda GC = 1.591) was primarily attributable to polygenic heritability (h^2^ = 0.3286 ± 0.0135) rather than population stratification (Intercept = 1.0176 ± 0.0169). This framework identified 2,212 significantly associated variants, encompassing 32 novel loci discovered via GWAS‐by‐Subtraction. Functional annotation indicated that the majority of these loci were located in intronic (57.92%) or intergenic (30.6%) regions. TWAS and fine‐mapping nominated 188 high‐confidence genes; SENP2 (TWAS Z = 12.5), KIF11 (Z = 11.5), and JAZF1 (Z = 11.38) exhibited the strongest positive associations with CKMs risk, whereas TCF7L2 (Z = −13.4) demonstrated the strongest inverse association. Pathway enrichment implicated lipid balance and ubiquitin‐mediated proteolysis, and cell‐type specificity analysis prominently localized genetic signals to pancreatic islet cells. Fine‐mapping prioritized 18 causal variants with posterior probability ⟩ 0.95, including key signals within GCKR (rs1260333, rs780093) and INADL (rs2481665). Chromosomal regions 7 and 12 exhibited significant contributions.

**Conclusions:**

Our study unravels the shared genetic architecture of CKMs, revealing novel risk loci and pathogenic mechanisms. The results establish a direct cellular link between the pleiotropic genetic basis of CKMs and endocrine metabolic regulation within pancreatic islets.

## Highlights

By integrating genomic structural equation modeling and transcriptomic analysis, we uncovered the shared genetic architecture of Cardiovascular‐Kidney‐Metabolic Syndrome (CKMs) and identified 32 novel risk loci. We revealed that these genetic signals are highly enriched in pancreatic islets and driven by intronic cis‐regulatory mechanisms modulating key genes like SENP2 and JAZF1. Ultimately, these insights provide a robust biological foundation to advance precision medicine and early‐stage targeted interventions.

## Graphical abstract

Flowchart illustration. BMI: Body mass index; FG: Fasting glucose; vitamin D: Serum 25‐Hydroxyvitamin D levels; CKD: Chronic kidney disease; CAD: Coronary artery disease; T2D: Type 2 diabetes.

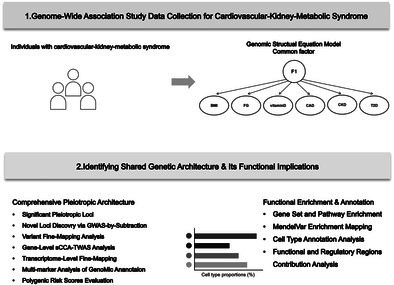

## 1 Introduction

Cardiovascular‐Kidney‐Metabolic Syndrome (CKMs) represents a complex, irreversible biological trajectory characterized by the progressive deterioration of multisystem metabolic homeostasis, deeply shaped by genetic, environmental, and lifestyle factors [1]. The accelerating aggregation of metabolic risk factors in populations has resulted in a rapid rise in CKMs incidence, posing substantial medical and socioeconomic challenges [2, 3]. Despite notable progress in mitochondrial‐nuclear genomic interactions over recent years, knowledge of the specific genetic and biological underpinnings of CKMs is still insufficient. Evidence suggests lipid metabolism as a major driver of CKMs [4], yet these insights alone cannot fully account for individual differences in disease progression and susceptibility.

In response to these challenges, this study integrates multiple genetic analytic tools and robust correlation‐exploration methods to elucidate underlying molecular mechanisms and broaden the links to various potential diseases. Particularly, we highlight genomic loci and chromosomal regions associated with CKMs to identify possible therapeutic intervention targets. Traditional univariate genome‐wide association studies (GWAS) have successfully identified numerous risk loci for individual cardiometabolic traits. However, they inherently fail to capture the latent genetic covariance and shared biological mechanisms driving the concurrent progression of multi‐organ dysfunction in CKMs. Therefore, Multivariate GWAS, such as Genomic Structural Equation Modeling (Genomic SEM) [5], could be a better‐suited method for investigating the broad genetic susceptibility across various phenotypes. Genomic SEM is a statistical modeling framework that integrates multivariate GWAS data, primarily decomposing the genetic covariance matrix of multiple phenotypes to create latent factors reflecting shared genetic architecture. By using the population covariance structure of gene‐level association signals, this approach simultaneously addresses pleiotropic genetic effects, adjusts for sample heterogeneity, resolves unknown sample overlap, and allows flexible structural equation modeling of causal pathways or latent biological pathway associations between phenotypes.

Additionally, it has been extensively applied to differentiate the shared genetic architecture across multiple diseases, ranging from psychiatric disorders [6] to immune diseases [7]. To address the lack of a unified genetic model for CKMs, we applied Genomic SEM to the summary statistics of six key cardiometabolic traits. By conceptualizing CKMs as a latent genetic factor, this approach allows us to associate specific SNPs with the broader CKMs phenotype, effectively overcoming the limitations of indirectly measured aspects of the syndrome. Furthermore, we integrated systems biology approaches—including transcriptome‐wide association studies (TWAS), fine‐mapping, and cell‐type enrichment—to translate these genetic signals into actionable biological insights. Ultimately, this research aims to bridge the gap between complex genomic statistics and basic research, providing a robust genetic architecture map of CKMs risk factors. By elucidating these shared molecular mechanisms, we seek to support the development of targeted, early‐stage clinical interventions and precision preventive strategies for this growing patient population.

## 2 Materials and Methods

### 2.1 Sources of Data for Single Input GWAS

The Single Input GWAS data related to CKMs in our study comes from six GWAS studies covering aspects such as type 2 diabetes, coronary artery disease, serum 25‐hydroxyvitamin D levels, BMI, chronic kidney disease, and fasting blood glucose. Each input GWAS was ethically approved by the relevant institutional review boards, and informed consent was obtained from all participants. The data underwent rigorous quality control. Detailed GWAS list information can be found in Table S1.

### 2.2 Quality Control for Single Input GWAS

1. Exclusion of low‐quality samples: Missing rate: Samples with a missing rate exceeding 5% are excluded.
2. MHC region (Major Histocompatibility Complex region, located on chromosome 6 (CHR = 6), with a genomic position approximately between 25 000 000 and 35 000 000 bp): Due to the genetic diversity and structural complexity of the MHC region, particularly the polymorphisms of immune‐related genes, special treatment is usually performed on the MHC region.
3. In preparing the summary statistics for the constructed CKMs, we used default parameters. We applied the recommended default quality control to filter all autosomal SNPs from the six input CKMs‐related GWAS, selecting SNPs based on the 1000 Genomes Phase 3 EUR panel. SNPs with MAF < 0.01 were excluded (as these SNPs are prone to errors due to limited samples in genotype clusters and have higher standard errors in LD score regression [LDSC]), SNPs with effect size estimates of zero were removed (to avoid affecting matrix reactivity, which is essential for Genomic SEM), and SNPs that did not match the reference panel or had allele mismatches were excluded [8].

### 2.3 Sample Overlap in Single Input GWAS

In our analysis, the Single Input GWAS data we incorporated came from various genomic data repositories, with participants differing between the datasets. This indicates that, in conducting the GWAS, we took the sample overlap between different cohorts into account to ensure both the accuracy and generalizability of the results, while also addressing the statistical effects of potential sample overlap. The extent of potential sample overlap was quantitatively assessed using the bivariate LDSC intercepts derived from the multivariable stage (Table S13).

### 2.4 Genomic Structural Equation Modeling

We employed the Genomic SEM available in the GenomicSEM R package (v.0.0.5) [5], to conduct a GWAS analysis on type 2 diabetes, coronary artery disease, serum 25‐hydroxyvitamin D levels, BMI, chronic kidney disease, and fasting blood glucose, investigating the underlying broad genetic susceptibility of these CKMs‐related traits. Genomic SEM is a newly developed approach that can examine multiple potential multivariate models to uncover the latent structure of the traits of interest. Detailed standards can be found in Table S2.

Genomic SEM is not influenced by sample overlap (e.g., UKB participants overlapping across multiple input GWAS) or biases caused by sample size imbalance. It also allows for the identification of variants that influence only certain complex traits, rather than all traits, which therefore do not represent broad cross‐trait susceptibility.

Genomic SEM is conducted in two phases. The first phase estimates the empirical genetic covariance matrix and the corresponding sampling covariance matrix. In the first stage, we prepared the summary statistics from the CKMs‐related GWAS and applied the multivariable extension of cross‐trait LDSC to generate the empirical genetic covariance matrix between the six traits, which served as input for the SEM common factor model. In the second stage, an SEM model is specified to minimize the hypothesized covariance matrix and the empirical covariance matrix calculated in the first phase. The main research goal here was to identify the genetic characteristics behind the six CKMs‐related traits; thus, we tested a single‐factor model. Model fit was assessed using SRMR, model *χ* ^2^, Akaike Information Criterion, and CFI. By applying the appropriate common factor SEM specifications, individual autosomal SNP associations were included in the genetic and related sample covariance matrices, generating a multi‐CKMs genome‐wide analysis result with shared covariance across 1 863 059 input CKMs‐related GWAS.

### 2.5 Genomic SEM SNP Heterogeneity

To evaluate whether SNP associations in CKMs are appropriately modeled within the multivariable structural equation model framework, we computed the SNP heterogeneity statistic. The null hypothesis of the SNP test is that the SNP association in a univariate phenotype GWAS is statistically mediated through CKMs. Hence, significant QSNP tests in CKMs suggest that the SNP acts through pathways beyond the shared genetic mechanisms of human metabolism as modeled by CKMs in order to assess QSNP heterogeneity (significance threshold *p* < 0.05).

### 2.6 Multilevel Assessment of Genomic SEM

LDSC genome‐wide control parameters were applied using a two‐step method (specific steps: no removal of SNPs with missing values, no removal of SNPs with INFO values below 0.9, no removal of SNPs with MAF [minor allele frequency] under 0.01, no removal of SNPs with *p* values outside the valid range, no removal of non‐SNPs or SNPs with ambiguous strand direction, removal of partitioned LD scores with zero variance. A two‐step estimator was used with a cutoff set at 30).

### 2.7 GWAS‐By‐Subtraction

In this study, we implemented the GWAS‐by‐subtraction approach by comparing the lead loci identified through the Genomic‐SEM CKMs latent factor with those reaching genome‐wide significance (*p* < 5 × 10^−8^) in the six single‐input GWAS. To ensure a robust subtraction, we masked all variants within a 500 kb window of the known lead SNPs that exhibited linkage disequilibrium (LD *r* ^2^ > 0.1), using the 1000 Genomes European panel as a reference. This approach effectively “subtracts” previously identified findings, thereby prioritizing 32 novel loci with high utility and precision for CKMs.

### 2.8 Defining Genomic Loci and Discovering Novel Variants

We applied FUMA (Functional Mapping and Annotation of GWAS) to identify genomic loci and determine the leading SNPs associated with CKMs [9], which were less correlated with other SNPs in linkage disequilibrium (LD < 0.1) and showed genome‐wide significance (*p* < 5 × 10^−8^). Initially, we entered the summary statistics of CKMs SNPs to evaluate their association strength.

We also compared the leading SNP loci with those found in the original univariate GWAS. To assess whether the 183 (leadSNPs in FUMA) leading SNPs in CKMs have pleiotropic associations, we also examined significant associations (*p* < 5 × 10^−8^) reported in the GWAS Catalog. Additionally, we conducted risk locus analysis of the CKMs model using FUMA software, with a significance threshold of (*p* < 5 × 10^−8^), and analyzed the corresponding output files using MAGMA (Multi‐marker Analysis of GenoMic Annotation), a tool used for post‐GWAS analysis to evaluate the relationship between genes and phenotypes (such as diseases or health traits). MAGMA combines multiple genetic markers (e.g., SNPs) into a gene‐level signal and assesses the association between each gene and the phenotype. Its purpose is to extract gene‐function related information from genome‐wide SNP data, enabling the analysis of gene‐level genetic signals, with a significance threshold of (FDR *p* < 0.05). Additionally, we developed an intriguing method (referred to as the GWAS reduction point method), where we compare the lead loci identified by the genomic structural equation model with those found through the genome‐wide significance threshold in univariate GWAS. This approach facilitates the discovery of novel and more valuable loci at the lead points.

### 2.9 Fine Mapping

To identify the most likely causal variants related to CKMs, we utilized SuSIE and FINEMAP [10], both of which are implemented in the R package echolocatoR v.2.0.3. We set the probability threshold at 0.95 to define the credible set of possible causal variants. The specific steps are as follows: Using SuSIE and FINEMAP to identify potential causal variants: SuSIE (Sum of Single Effects) and FINEMAP are tools for fine‐mapping analysis aimed at identifying the most probable causal variants associated with a phenotype. In this step, we applied a 250 kb window to cover the region associated with each lead SNP and calculated the causal inference probability for each SNP within these regions. Credible set: We established a probability threshold of 0.95, and if a variant's posterior probability (PP) exceeds this threshold, it is regarded as a potential causal variant. Consensus SNPs and probability set: echolocatoR defines a “consensusNP,” meaning variants that appear in both SuSIE and FINEMAP results. For these consensus SNPs, the tool calculates their average PP and determines the average credible set based on the probability outcomes (when the posterior probabilities of SNPs from SuSIE and FINEMAP exceed 0.95, the credibility is set to 1; otherwise, it is set to 0).

### 2.10 Transcriptome‐Wide Association Study (TWAS)

After pinpointing potential causal variants, we performed a cross‐tissue sCCA‐TWAS (sparse canonical correlation analysis‐based Transcriptome‐Wide Association Study) [11], designed to prioritize genes associated with CKMs based on the relationship between gene expression and phenotype. We used the FUSION method for TWAS, and the pre‐calculated 37 920 expression quantitative trait loci (eQTL) features from the GTEx v.8 data (i.e., gene/tissue pairs). These features are used to compute the expression associations between various genes and tissues. This approach aims to pinpoint genes most relevant to the Genomic‐SEM of CKMs by leveraging 37 920 precomputed eQTL features from the GTEx (v8) dataset. We then selected genes with FDR‐corrected TWAS *p* values < 0.05 for further analysis using fine‐mapping of gene sets (FOCUS), which evaluates the likelihood of a causal gene–phenotype relationship based on the FOCUS posterior inclusion probability. FOCUS applies a Bayesian framework to probabilistically attribute causality to genes associated with GWAS signals. By integrating multi‐omics data, it addresses pleiotropy and LD confounding, calculating posterior inclusion probabilities (PIPs) to quantify the likelihood of a gene's causal role (Table 1).

**TABLE 1:** Genetic associations with CKMs in sCCA and FOCUS analysis.

Gene	Heritability squared	TWAS *Z*	TWAS FDR *P*	CHR	Srart position	End position	FOCUS pip	Tissue
SENP2	0.0811	12.50546	9.92E−32	3	185 582 496	185 633 551	1	Artery—Coronary
KIF11	0.1104	11.5	1.41E−26	10	92 593 286	92 655 395	1	Whole blood
JAZF1	0.2264	11.381616	3.67E−26	7	27 830 573	28 180 743	1	Pancreas
JAZF1	0.3417	11.12502	5.39E−25	7	27 830 573	28 180 743	1	Liver
JAZF1	0.234	9.03131	6.05E−16	7	27 830 573	28 180 743	1	Adipose—Visceral (omentum)
LMOD1	0.3392	8.644493	1.40E−14	1	201 896 452	201 946 588	1	Artery—Aorta
LMOD1	0.2399	8.39117	1.14E−13	1	201 896 452	201 946 588	1	Heart—Left ventricle
CDKAL1	0.0825	8.1315	9.28E−13	6	20 534 457	21 232 404	1	Pancreas
AC010883.5	0.2518	7.7	2.01E−11	2	43 229 573	43 233 394	0.998	Brain—Cortex
UBE2Z	0.1013	7.6428	2.53E−11	17	48 908 369	48 929 056	1	Liver
NRBP1	0.3226	7.602398	3.31E−11	2	27 427 790	27 442 259	1	Whole blood
LMOD1	0.2793	7.46362	7.73E−11	1	201 896 452	201 946 588	1	Esophagus—Muscularis
NRBP1	0.3195	7.25	3.06E−10	2	27 427 790	27 442 259	0.999	Spleen
RP11‐51F16.9	0.05	7.23703	3.26E−10	17	63 610 772	63 611 184	0.982	Pituitary
COG8	0.1222	7.2028	3.82E−10	16	69 320 140	69 339 429	0.811	Pancreas
MACF1	0.181	6.908742	2.48E−09	1	39 081 316	39 487 177	1	Brain—Cortex
PMAIP1	0.103	6.842901	3.74E−09	18	59 899 948	59 904 306	1	Whole blood
PLEKHH2	0.3985	6.818653	4.36E−09	2	43 637 273	43 767 987	0.981	Kidney—Cortex
C18orf8	0.3835	6.67494	1.12E−08	18	23 503 470	23 530 612	0.989	Liver
NRBP1	0.0471	6.610806	1.56E−08	2	27 427 790	27 442 259	0.999	Small intestine—Terminal ileum
LPL	0.3233	6.50916	2.66E−08	8	19 901 717	19 967 258	1	Muscle—Skeletal
KAT6A	0.088	6.30796	8.30E−08	8	41 929 479	42 051 990	0.951	Whole blood
CEP68	0.521	6.000237	4.18E−07	2	65 056 366	65 086 854	0.996	Spleen
CEP68	0.6182	−6.01	4.04E−07	2	65 056 366	65 086 854	0.991	Whole blood
CAMK2G	0.0902	−6.066851	2.91E−07	10	73 812 501	73 874 591	0.805	Brain—Cerebellum
SOX7	0.0928	−6.15116	1.90E−07	8	10 723 768	10 730 512	0.988	Artery—Aorta
ANKDD1B	0.2256	−6.189762	1.56E−07	5	75 611 459	75 671 846	0.999	Pituitary
UBE2E2	0.0719	−6.316995	7.91E−08	3	23 203 020	23 591 793	1	Pancreas
RBMS1	0.0993	−6.32	7.91E−08	2	160 272 151	160 493 794	1	Adipose—Visceral (omentum)
RP11‐574 K11.24	0.1869	−6.38196	5.66E−08	10	73 742 995	73 744 230	0.789	Thyroid
CDKN2B	0.1383	−6.505	2.70E−08	9	22 002 903	22 009 363	1	Artery—Tibial
AC010883.5	0.292	−6.521892	2.50E−08	2	43 229 573	43 233 394	1	Brain—Frontal cortex (BA9)
ARG1	0.0712	−6.60612	1.56E−08	6	131 573 144	131 584 332	1	Liver
BECN1	0.3632	−6.87574	3.08E−09	17	42 811 655	42 833 141	0.768	Kidney—Cortex
MACF1	0.1802	−7.01448	1.24E−09	1	39 081 316	39 487 177	1	Adipose—Visceral (omentum)
CELF1	0.1465	−7.2225	3.46E−10	11	47 465 944	47 565 335	0.986	Muscle—Skeletal
NFAT5	0.1894	−7.39541	1.15E−10	16	69 565 094	69 704 666	1	Kidney—Cortex
ANK1	0.2434	−7.40214	1.13E−10	8	41 653 220	41 896 762	1	Whole blood
UBE2Z	0.1166	−7.64276	2.53E−11	17	48 908 369	48 929 056	0.707	Pancreas
DCAF7	0.1171	−7.66286	2.35E−11	17	63 550 461	63 594 266	0.805	Muscle—Skeletal
FTO	0.2755	−7.6822	2.11E−11	16	53 701 692	54 158 512	1	Brain—Hypothalamus
PTPMT1	0.1641	−7.71467	1.91E−11	11	47 565 797	47 572 196	0.956	Heart—Left ventricle
WDR59	0.0829	−7.8695	6.26E−12	16	74 871 367	75 000 173	1	Pancreas
E2F3	0.1147	−8.782057	4.58E−15	6	20 401 906	20 493 715	1	Whole blood
ALKAL2	0.1305	−8.90464	1.69E−15	2	279 558	288 851	1	Adrenal gland
TRA2B	0.0704	−9.441293	1.50E−17	3	185 915 906	185 938 136	1	Whole blood
TCF7L2	0.1435	−13.4	1.19E−36	10	112 950 250	113 167 678	1	Pancreas

### 2.11 Gene Set and Disease Ontology Enrichment Analysis

We used MAGMA and FUMA (GESA) data for gene enrichment analysis and gene pathway set analysis, exploring the potential relationship between CKMs and Mendelian disease genes and their related pathways. Furthermore, we performed gene enrichment analysis using MendelVar (https://mendelvar.mrcieu.ac.uk/submit/).

### 2.12 Cell Annotation Analysis

To identify the etiological cell types related to CKMs, we employed CELLECT [12], which integrates cell‐type‐specific expression for complex traits using single‐cell RNA sequencing data. We utilized the Tabula Muris [13] dataset, which includes transcriptomic data from 100 000 mouse ( *Mus musculus* ) cells from 20 organs and tissues. We performed preprocessing and standardization of the single‐cell RNA sequencing data from Tabula Muris using CELLEX, and calculated the expression specificity probability scores for each gene. We used the LDSC software for cell‐type specificity analysis, categorized the cell types, and applied a false discovery rate (FDR) threshold of 0.05.

### 2.13 Genetic Contribution of Genomic Regions

The LDSC tool computes the partitioned heritability of the genome. It assesses the contribution of each genomic region to phenotypic heritability by assigning the genetic information of the phenotype to various genomic regions (such as genes, enhancers, silencers, etc.). Specifically, LDSC employs a weighted LD matrix, genotype frequency files, and summary statistics for the calculation, estimating the genetic contribution of each region.

### 2.14 Construction of Polygenic Risk Scores Based on Summary Data

We calculated polygenic risk scores (PRS) from genome‐wide summary statistics and assessed the genetic contribution of various chromosomal regions to disease onset. Specifically, the method employed PRS‐CS (Polygenic Risk Score with Continuous Shrinkage) software to estimate the posterior effect values of SNPs and calculate the PRS using GWAS data and an external LD reference panel. This method employs a Bayesian regression model, which estimates effect values by integrating the LD reference panel based on GWAS summary statistics and subsequently calculates the PRS.

## 3 Results

### 3.1 Genetic Correlations and Model Fit

**FIGURE 1:**
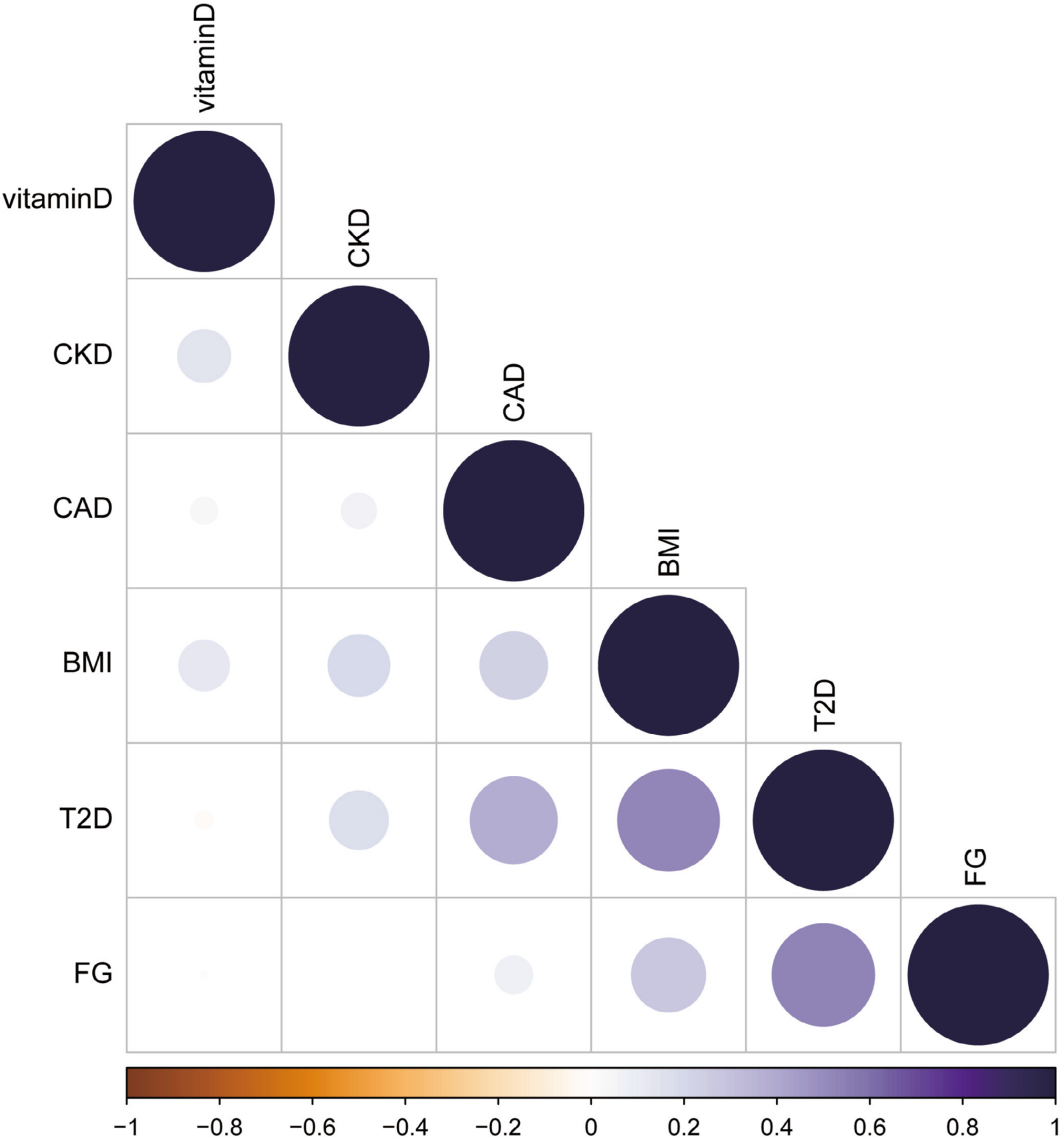
Genetic correlations for SEM with genomic SEM, displaying pairwise LD score genetic correlation estimates for the six univariate phenotypes.

LD‐Score regression analysis [14] derived SNP‐based heritability (*h* ^2^SNP) estimates for the six univariate input GWAS traits: type 2 diabetes (T2D) (19.5%), coronary artery disease (CAD) (14.7%), serum 25‐hydroxyvitamin D levels (25 (OH)D) (9.39%), body mass index (BMI) (28.2%), chronic kidney disease (CKD) (3.11%), and fasting glucose (FG) (6.36). We employed genomic SEM using the genetic covariance matrix. The genetic covariance values between each pair of traits are presented in Figure 1. The common factor model exhibited strong fit to the empirical covariance matrix, as evidenced by a Comparative Fit Index (CFI) of 0.99 and a Standardized Root Mean Square Residual (SRMR) of 0.14 (Table S3). Estimates of the latent variable loadings and residual covariances are provided in Table S4. Factor loadings and residual covariances indicated significant shared genetic factors among these traits. After excluding single‐nucleotide polymorphisms (SNPs) demonstrating substantial heterogeneity (Q *p* value < 0.05), the final genomic SEM analysis characterized the genetic architecture of the CKMs using 1 862 425 high‐quality SNPs.

### 3.2 Genomic Control and Signal Inflation Assessment

**FIGURE 2:**
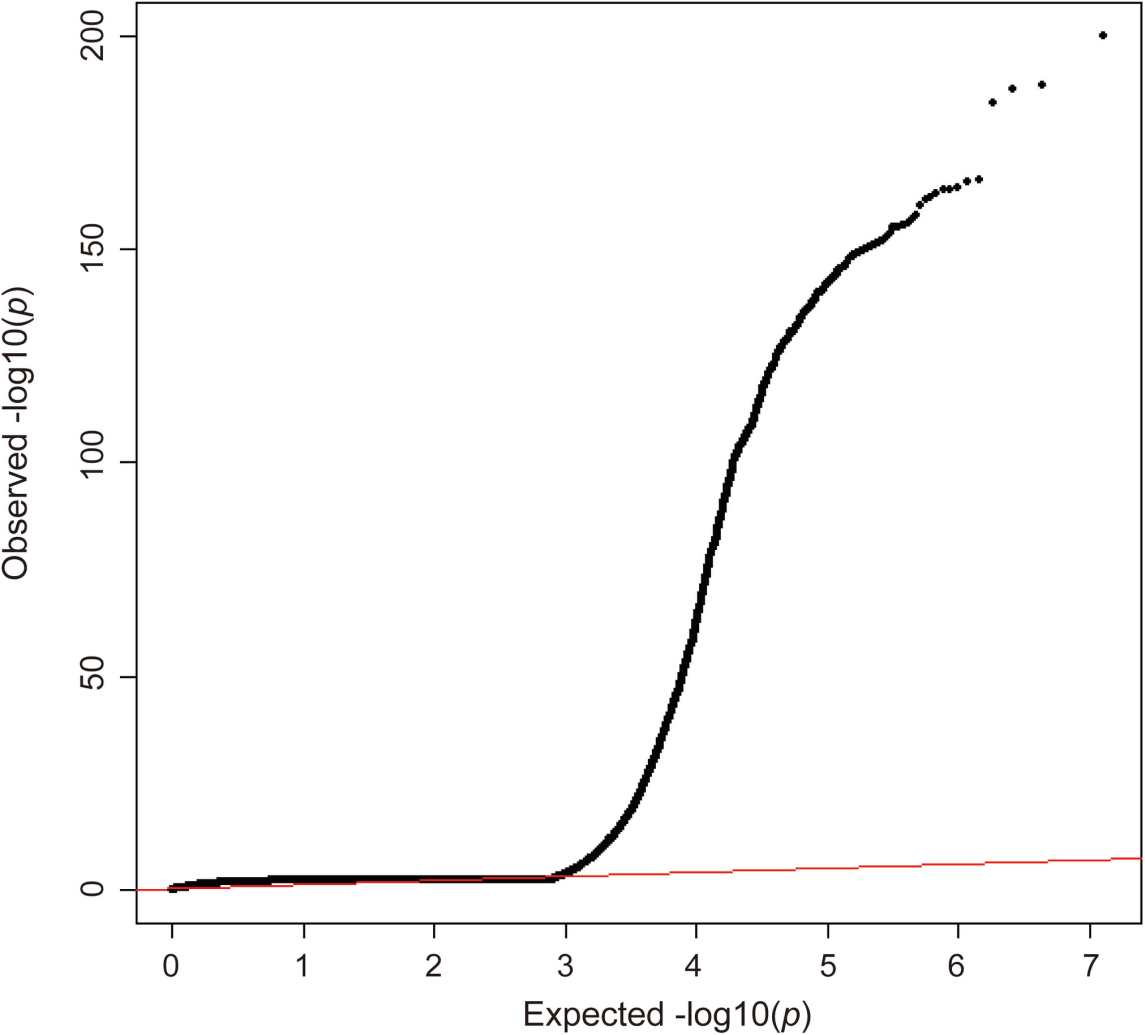
QQ plot of GWAS summary statistics for CKMs.

To assess the origins of inflation in structural equation models, we optimized method parameters to filter genetic variants. Specifically, we excluded 1 025 239 SNPs during quality control, resulting in 837 184 robust SNPs after regression coefficient application. Statistical evaluations demonstrated a mean Chi^2^ value of 1.897, a genomic control Lambda GC of 1.591, and an extreme Chi^2^ maximum of 913.763. While these values indicate substantial genomic inflation, further analysis confirmed that this originates from true polygenic signals rather than bias. Specifically, the LDSC intercept stood at 1.0176 (SE = 0.0169), which is effectively close to 1.0. Additionally, the LDSC ratio was measured at 0.0196 (SE = 0.0189), suggesting that over 98% of the observed inflation is attributable to total heritability (*h* ^2^ = 0.3286, SE = 0.0135) rather than population stratification or sample overlap. Furthermore, the cross‐trait bivariate LDSC intercepts confirmed that the sample overlap and cryptic relatedness among the six source GWAS datasets were negligible (Table S13). Heterogeneity tests yielded non‐significant results (*p* > 0.05). Collectively, these multiple estimates converge to indicate that the phenotypic inflation is driven by robust polygenic heritability signals. The characteristic tail inflation is visually supported by the QQ plot provided in Figure 2.

### 3.3 Identification of Novel Risk Loci and Functional Annotation

**FIGURE 3:**
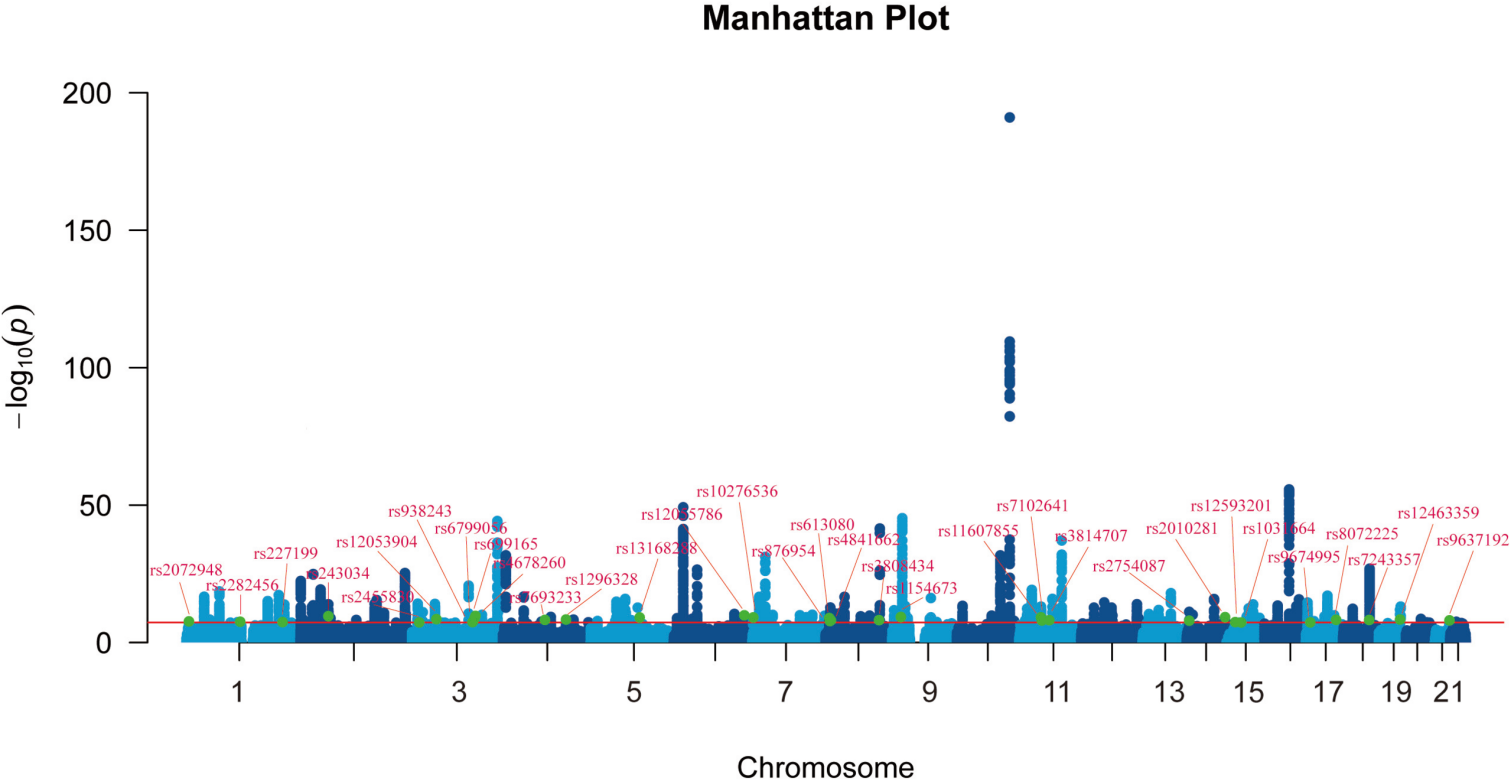
Manhattan plot of novel genomic‐SEM results for CKMs. The x‐axis denotes chromosomal positions, while the y‐axis represents the negative logarithm of the *p* value (−log10 (*P*)). The red line indicates the genome‐wide significance threshold at *p* = 5 × 10^−8^. Labeled points correspond to novel SNPs identified through the novel GWAS approach.

To discover novel genetic associations for complex traits, we implemented a genomic‐SEM framework, analyzing 1 025 239 SNPs. This identified 2212 genome‐wide significant variants (*p* < 5 × 10^−8^), confirming robust statistical significance. Applying the GWAS‐by‐Subtraction method, we contrasted findings against established datasets, revealing 32 unreported loci absent from the original GWAS summaries or GWAS Catalog (Figure 3, Table S5). Functional annotation with FUMA showed that these variants were concentrated in intronic (57.92%) and intergenic (30.6%) regions, while only 9 SNPs resided in exonic sequences. As is highly characteristic of complex polygenic traits, this predominant localization in non‐coding regions indicates that these variants primarily influence CKMs susceptibility not by altering protein structure, but rather by functioning as cis‐regulatory elements that modulate downstream gene expression and RNA splicing. Subsequent analysis yielded 420 independent SNPs (*r* ^2^ < 0.6) and 183 lead variants (*r* ^2^ < 0.1) (Table S6), delineating 151 distinct risk loci (Table S7). Previous studies show that rs2481665 plays a role in insomnia [15], but this locus is not directly responsible for CKMs; rather, it is a potential mediator locus. rs12088739 has been found to be consistent with our phenotype in most prior studies [16]. Gene‐based assessment via MAGMA pinpointed 118 potential CKMs‐related genes. Functional annotation further indicated that 111 candidate genes (94.1%) encompassed genomic regions with high predicted pathogenicity (CADD Scaled C‐Score ≥ 10), including 34 (28.8%) harboring extremely deleterious variants (CADD ≥ 20), underscoring their pathogenic impact (Table S8, Figure S1).

### 3.4 Fine‐Mapping and Causal Variant Prioritization

**FIGURE 4:**
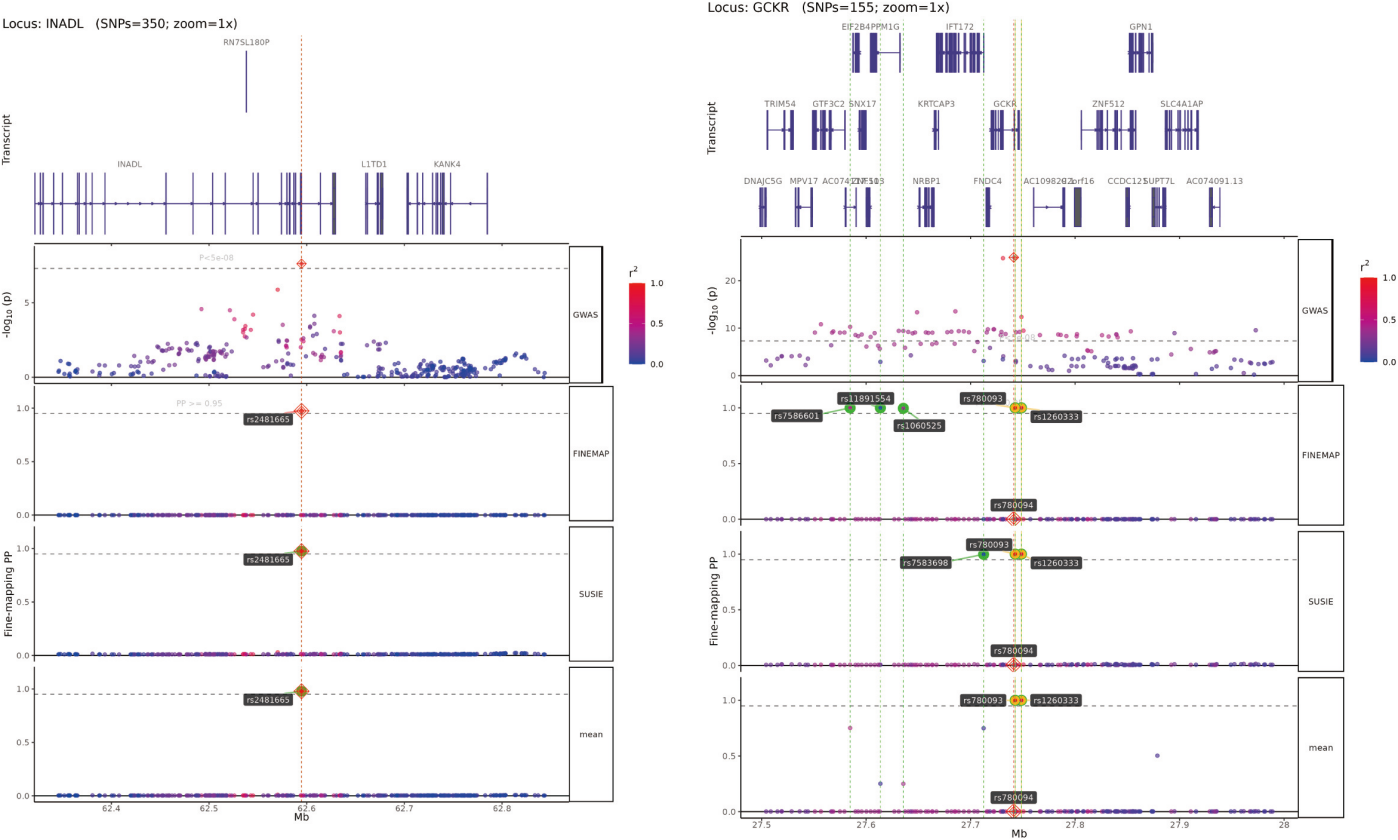
Fine‐mapping results of genomic loci with strong associations (PP > 0.95) identified by FINEMAP.

Fine‐mapping analyzes identified 18 genomic loci achieving both a PP threshold > 0.95 and genome‐wide significance (*p* < 5 × 10^−8^). Key associations included rs1260333 and rs780093 mapping to GCKR (chromosome 2), and rs2481665 in INADL (chromosome 1). Regional plots demonstrated distinct signal peaks at these loci, supported by additional credible‐set variants establishing trait associations (Figure 4).

### 3.5 Transcriptome‐Wide Association and Gene Prioritization

**FIGURE 5:**
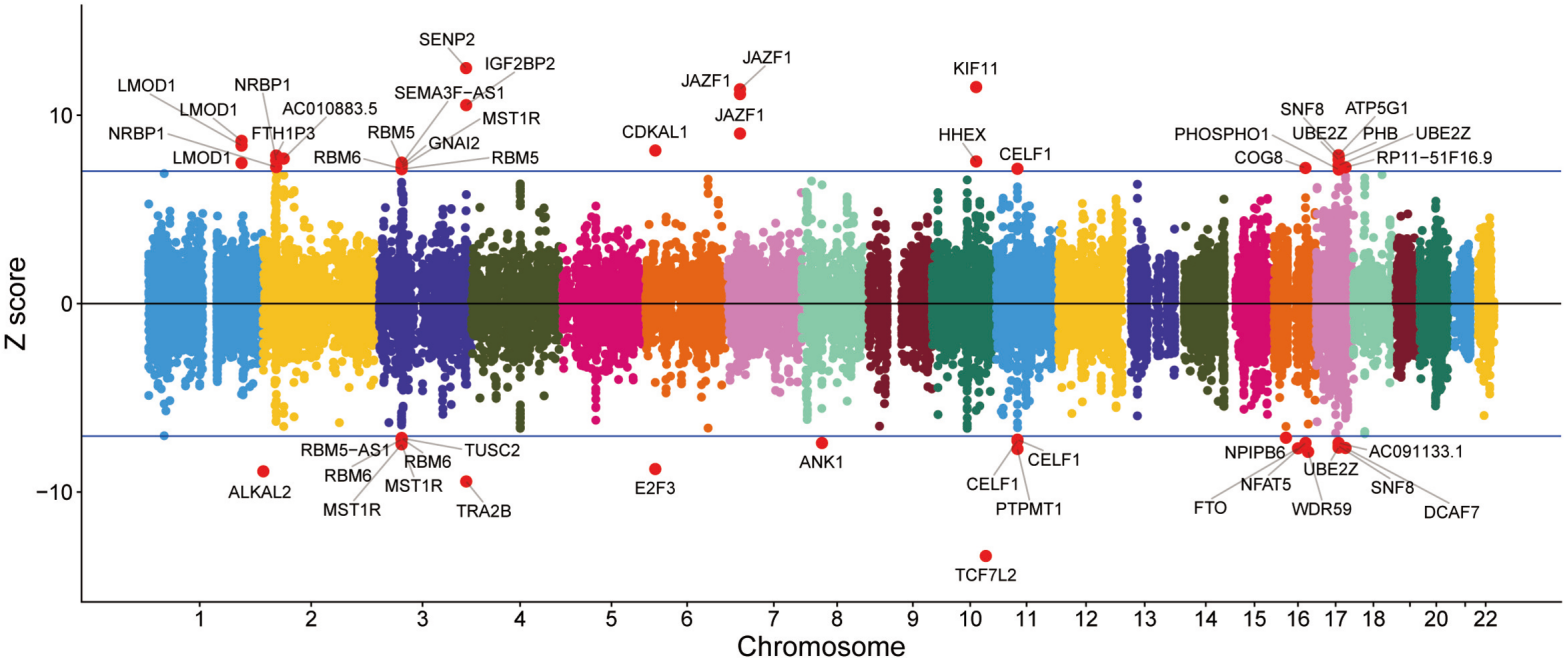
Manhattan plot of the TWAS *Z*‐scores for CKMs. The x‐axis represents chromosomes, and the y‐axis displays the *Z*‐scores. The horizontal blue lines mark the absolute *Z*‐score value of 6, which represents the threshold for significance.

To elucidate gene‐level associations underlying CKMs pathogenesis, we performed transcriptome‐wide association analysis (TWAS) using FUSION. This identified 228 genes passing multiple comparison correction, including 69 with TWAS *Z*‐scores > 6 (Figure 5). Subsequent fine‐mapping via FOCUS revealed 188 candidate genes potentially associated with CKMs pathogenic signals. Intersection analysis validated these high‐confidence associations: SENP2 exhibited the strongest positive *Z*‐score (12.5), followed by KIF11 (11.5) and JAZF1 (11.38), collectively indicating elevated predicted expression correlates with CKMs susceptibility. Conversely, TCF7L2 displayed the highest inverse association (*Z* = −13.4), trailed by TRA2B (*Z* = −9.44), suggesting reduced expression enhances disease risk.

### 3.6 Cell‐Type Specificity and Functional Pathway Enrichment

A primary and highly relevant finding of our study is the profoundly significant enrichment of CKMs genetic signals within pancreatic islet cells. Across diverse cell types, enrichment analysis revealed seven cell types surpassing the significance threshold after multiple comparison corrections (Table S10), with the Pancreatic Islets and Limbic System ranked as the top two; notably, the pancreatic islets exhibited the most significant enrichment of CKMs. This finding directly anchors the pleiotropic genetic basis of CKMs to a key endocrine cell type.

**FIGURE 6:**
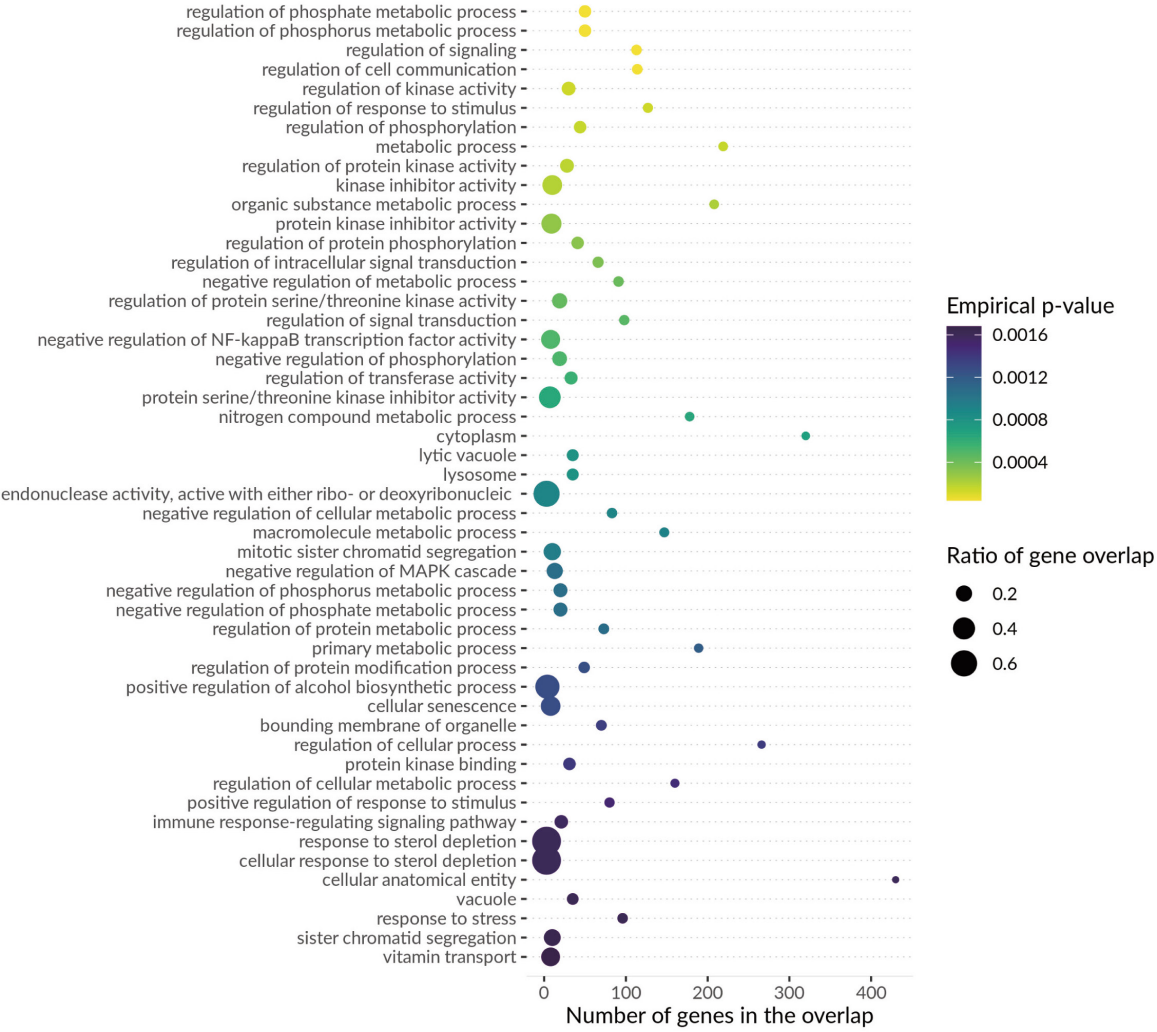
Bubble plot of biological processes from MendelVar enrichment mapping.

Consistent with this cellular context, we employed multi‐marker genomic annotation analysis (MAGMA) and identified 262 genes that were subsequently analyzed using gene set enrichment analysis (GSEA). This analysis demonstrated significant enrichment in GSEA entries associated with lipid balance, ubiquitin‐mediated proteolysis, and trypsin functions, including roles in regulating hormone levels and protein secretion (Table S9). Furthermore, GSEA confirmed biological processes enriched by MendelVar, such as kinase activity regulation and protein phosphorylation regulation (Figure 6). Collectively, these findings suggest that the coordinated dysregulation of metabolic pathways within pancreatic islets serves as a central hub in the pathogenesis of CKMs.

### 3.7 Genetic Contribution of Genomic Regions

Analysis of genetic contributions from Table S11 revealed that significant contribution sites are highly enriched in regulatory regions—specifically promoters, enhancers, and gene‐dense areas—essential for gene expression regulation, chromatin modification, and transcription factor binding. Promoter and enhancer regions exhibited the most pronounced effects, with genetic variations substantially altering gene expression to impact trait or disease susceptibility. Substantial contributions were also detected in select non‐coding regions, suggesting their involvement in complex genetic mechanisms through expression or functional regulation.

### 3.8 Contributions at the Chromosome Level

We analyzed the association between pre‐constructed PRS variant sites and disease susceptibility, assessing the relative contributions of specific chromosomal regions. The findings demonstrate significant heterogeneity in the magnitude of genetic contributions across chromosomal regions. Notably, regions on chromosomes 7 and 12 contribute disproportionately to disease susceptibility compared to other regions. This suggests these loci likely harbor critical genes or regulatory elements involved in disease pathogenesis (Table S12).

## 4 Discussion

This study thoroughly explores the genetic basis of type 2 diabetes, coronary artery disease, serum 25‐hydroxyvitamin D levels, BMI, chronic kidney disease, and fasting blood glucose, utilizing various approaches, including Genomic SEM, summary data PRS, MR‐IEU, fine mapping, and transcriptomic analysis. The clinical interconnectivity of these conditions was recently formalized by the American Heart Association's definition of CKMs, which emphasizes the shared pathophysiology among obesity, diabetes, chronic kidney disease, and cardiovascular disease [17]. Aligning with this clinical framework, our Genomic SEM analysis confirmed that these phenotypes are driven by substantial genetic covariance, indicating that CKMs is a unified genetic entity. By employing a GWAS‐by‐subtraction approach, we identified 2212 genome‐wide significant variants associated with the shared CKMs factor, including 32 previously unreported loci, demonstrating how multivariate genetic models can effectively complement traditional GWAS in studying complex, multi‐organ conditions [18].

Consistent with the underlying architecture of complex multi‐trait phenotypes, functional annotation revealed that 57.92% of the significant loci associated with CKMs are situated in intronic regions. While this distribution is a typical characteristic of genome‐wide association studies for complex traits [19], the dominance of intronic variants in CKMs genetics underscores the fundamental role of cis‐regulatory mechanisms in driving multisystem disease risk. These non‐coding regions are heavily enriched in critical regulatory elements, including enhancers, silencers, and splicing branchpoints, which function as molecular switches to modulate gene dosage [20]. Our integration of GTEx v8 eQTL data [21] robustly confirms this mechanistic drive, demonstrating that the expression levels of our prioritized TWAS genes—specifically SENP2, KIF11, and JAZF1—are predominantly modulated by local cis‐eQTL variants. This functional convergence strongly suggests that the non‐coding GWAS signals (such as those predominant intronic variants) primarily influence CKMs susceptibility by perturbing cis‐regulatory networks, rather than altering protein‐coding sequences. These regulatory variants likely influence the affinity of transcription factor binding sites or modify chromatin accessibility, thereby fine‐tuning the transcriptional output of master metabolic regulators [22]. Furthermore, our regional contribution analyzes highlighted the disproportionate pathophysiological contributions of specific chromosomes, particularly novel regulatory regions on chromosomes 7 and 12, which harbor risk loci linked to both eQTLs and long non‐coding RNA (lncRNA) expression [23]. Collectively, these findings validate that CKMs is mechanistically driven by the intricate, tissue‐specific regulatory tuning of gene expression networks and the orchestration of the non‐coding transcriptome, providing a rigorous biological link between intronic variation and the synchronized deterioration of cardiovascular and metabolic health.

A primary advancement of our study is the prioritization of novel actionable targets for precision medicine via TWAS [24]. Following rigorous Benjamini‐Hochberg false discovery rate (FDR) correction, our analysis revealed strong, novel multisystem‐level associations for SENP2, KIF11, and JAZF1. Importantly, our cross‐tissue integration provided specific physiological contexts for these associations. Among these, SENP2 has been functionally characterized in depth. SENP2, a crucial SUMO‐specific protease, regulates the SUMOylation of essential transcription factors and thereby governs the metabolic adaptability of brown adipose tissue [25]. Studies have confirmed that SENP2‐dependent deSUMOylation enhances fatty acid oxidation and insulin secretion to boost thermogenesis [26]; mice lacking SENP2 specifically in brown adipose tissue display cold intolerance and insulin resistance [27]. The functional variants uncovered here might interfere with SENP2 enzymatic function, disturbing the SUMOylation homeostasis of metabolic regulators (e.g., PPARγ, LXR), consequently causing hepatic lipid metabolic disorders, adipose tissue impairment, and reduced systemic insulin sensitivity—thus offering a potential pathophysiological explanation for CKMs. Furthermore, KIF11, identified predominantly in whole blood, showed a notable association in our model. KIF11 functions as a motor protein essential for mitotic spindle formation. Research indicates that KIF11 is crucial for vascular smooth muscle cell proliferation, where its overexpression can accelerate neointimal formation after vascular injury [28]. While KIF11's mitotic role is well characterized, emerging genetic evidence in CKMs highlights an unrecognized contribution to metabolic stability, providing a novel perspective on how KIF11 bridges metabolic and vascular pathways. JAZF1, another candidate gene identified, is a gene that encodes a member of the zinc finger protein family. Moreover, JAZF1 emerged as a molecular hub primarily active within the pancreas and visceral adipose tissue. JAZF1 is known to modulate metabolism by regulating lipid homeostasis and reducing inflammatory responses in metabolic tissues [29]. GWAS findings indicate that genetic polymorphisms in JAZF1 are strongly linked to the risk of type 2 diabetes (T2DM). Both diabetic patients and obese animal models exhibit markedly reduced expression of JAZF1. Mice lacking JAZF1 show phenotypes of delayed growth, insulin resistance, and β‐cell impairment [30]. Our study provides the first multisystem‐level evidence for JAZF1's integrative role in CKMs. Findings suggest that JAZF1 functions not merely as a risk gene for metabolic disorders but also as a key molecular hub bridging energy metabolism dysregulation and cardiac remodeling.

In contrast to the genes discussed above, TCF7L2—an extensively investigated T2DM susceptibility gene [31]—was found to be significantly inversely associated with CKMs risk in our analysis. Earlier evidence demonstrated that polymorphisms in TCF7L2, such as rs7903146, influence insulin secretion by pancreatic β‐cells and regulate glucose homeostasis [32]. The present findings imply that higher expression of TCF7L2 may confer protective effects against CKMs by preserving insulin output and metabolic equilibrium.

At the cellular levels, we identified a profoundly significant enrichment of CKMs genetic signals within pancreatic islet cells. This finding directly anchors the pleiotropic genetic basis of CKMs to the primary endocrine cell type responsible for systemic metabolic homeostasis, aligning with recent single‐cell transcriptomic insights into metabolic dysregulation [33]. Our sCCA and subsequent FOCUS fine‐mapping [34] identified 31 genes with potential causal roles in CKMs. These genes are primarily involved in crucial biological pathways such as lipid balance and ubiquitin‐mediated proteolysis and are strongly linked to established disease pathways like type 2 diabetes, metabolic syndrome, and myocardial infarction. For instance, key genes in the lipid balance pathway could increase an individual's susceptibility to metabolic diseases, such as diabetes and atherosclerosis, by influencing processes like insulin resistance, chronic inflammation, oxidative stress, and endothelial dysfunction [35, 36].

Furthermore, the ubiquitin‐proteasome system (UPS) is crucial for maintaining intracellular protein homeostasis, regulating signal transduction, and controlling gene expression [37]. UPS dysregulation causes the abnormal accumulation of misfolded proteins (such as troponin) in cardiomyocytes, triggering endoplasmic reticulum stress and mitochondrial dysfunction, thereby promoting the development of cardiovascular diseases [38]. Similarly, in the renal system, this proteolytic network precisely regulates protein homeostasis, playing an essential role in maintaining the integrity of the glomerular filtration barrier and the survival of renal tubular epithelial cells [39].

While our pathway analysis revealed an enrichment in trypsin‐associated biological processes, it is important to clarify that none of our prioritized causal genes encode classical exocrine digestive proteases. Instead, within the multisystem pathophysiological context of CKMs [17], this enrichment acts as a robust transcriptomic proxy for generalized extracellular proteolytic dysregulation and matrix remodeling [40]. Crucially, this systemic proteolytic signature is tightly linked to our actual genetic findings through its profound functional coupling with the intracellular UPS [41]. This intrinsic connection is directly anchored by our top prioritized candidate, SENP2, a SUMO‐specific protease that dictates the stability and UPS‐mediated degradation of essential metabolic transcription factors [42]. Furthermore, this widespread extracellular proteolytic stress and matrix remodeling provide the necessary microenvironmental scaffolding for active endothelial proliferation and structural vascular alterations driven by essential motor proteins like KIF11 [43]. Concurrently, it creates a hostile, inflammatory microenvironment that directly antagonizes the protective, lipid‐modulating functions of JAZF1 and exacerbates the profound metabolic dysfunction associated with TCF7L2 variants [44]. Thus, the co‐enrichment of these proteolytic, UPS, and lipid pathways reflects a synergistic pathogenic axis—mechanistically grounded entirely by our core identified genes—that underpins the multi‐organ deterioration in CKMs.

Although this study provides new insights into the genetics of CKMs, there are still several limitations. First, our findings are primarily derived from data of European ancestry (the 1000 Genomes EUR panel), which limits the generalizability of our conclusions to other ancestral populations. Given the well‐established ethnic disparities in CKMs‐related traits, caution is warranted when extrapolating our results to a global context. In future work, we plan to validate the top loci identified in this study within more diverse cohorts (e.g., African or Asian) to assess their trans‐ethnic portability and to investigate potential ancestry‐specific genetic architectures. Secondly, although we have identified several gene loci associated with CKMs through fine mapping and transcriptomic analysis, linking these genes to specific biological mechanisms remains an unresolved challenge. Future studies should further explore how these genetic variations affect gene expression, neurodevelopment, and metabolic pathways. Thirdly, we have not sufficiently considered the influence of environmental interactions (such as diet, exercise, and lifestyle habits) on CKMs in the current version of the analysis. This limitation stems from the lack of comprehensive environmental data, as well as the potential changes in outcomes when such factors are reintroduced into the analysis. Although environmental factors may play a crucial role in regulating the gene‐phenotype relationship, we did not conduct the relevant analysis in this study due to these limitations. In future research, we aim to incorporate more comprehensive environmental data to explore how gene‐environment interactions affect the pathogenesis of CKMs. Lastly, a limitation of this study is the statistical power issue concerning low‐heritability traits (such as CKD, *h* ^2^ = 3.11%). Because CKD has low heritability, its genetic signals are relatively weak, which may lead to inadequate statistical power when performing genome‐wide association studies using standard methods. To address this issue, we plan to utilize larger sample sizes and advanced statistical techniques in future studies. As a result, although we utilized large‐scale datasets and applied multiple statistical methods, some potentially significant genetic variations may have gone undetected due to the weak genetic signals. We aim to improve the sensitivity of our analysis by incorporating more robust tools to identify these variations. In future studies, we intend to use larger sample sizes and integrate additional analytical tools to further improve statistical power and the reliability of the results.

## 5 Conclusion

This study provides new insights into the genetic foundation of CKMs. By integrating Genomic SEM, fine mapping, and transcriptomic analysis, we identified several new genetic loci and demonstrated how these loci influence the genetic links between gene expression and complex traits. Our findings not only enhance the understanding of the genetic mechanisms underlying CKMs but also offer new ideas for precision medicine and public health interventions. Future studies will further validate these genetic markers and investigate the role of gene‐environment interactions in multi‐organ metabolic homeostasis, with the goal of improving global healthy life expectancy and quality of life.

## Author Contributions

C.L.: writing – original draft, writing – review and editing. L.L.: data curation, investigation, writing – review and editing, methodology. J.C.: formal Analysis, writing – review and editing. R.C.: investigation, writing – review and editing. H.D.: supervision, writing – original draft.

## Funding

The authors have nothing to report.

## Ethics Statement

This study utilized publicly available data and therefore did not require ethical approval.

## Conflicts of Interest

The authors declare no conflicts of interest.

## Supporting information


**Figure S1:** Manhattan plot of GWAS results for CKMs from MAGMA analysis.

**Table S1:** GWAS summary sources.
**Table S2:** SNP heritability of genomic‐SEM phenotypes.
**Table S3:** Model fit indices for the genomic structural equation model.
**Table S4:** Factor loadings and residual covariances in genomic structural equation model of CKMs.
**Table S5:** Novel SNP variants identified by genomic‐SEM.
**Table S6:** Lead SNP identified by genomic‐SEM.
**Table S7:** Risk locus identified by genomic‐SEM.
**Table S8:** MAGMA risk gene annotation using genomic‐SEM.
**Table S9:** Enriched pathways by MsigDB.
**Table S10:** Enriched cell types in GWAS for CKMs.
**Table S11:** Heritability enrichment across genomic functional and regulatory regions.
**Table S12:** Polygenic risk score and genetic contribution across chromosomal regions.
**Table S13:** Estimates of sample overlap and cryptic relatedness across the six source GWAS traits.

## Data Availability

The data that supports the findings of this study are available in the Supporting Information of this article.
