# Supplementary figures and images for: Genomic Structural Equation Modeling Reveals Cardiovascular‐Kidney‐Metabolic Syndrome Genetic Architecture
Source: J Diabetes. 2026 Apr 13;18(4):e70225. doi: 10.1111/1753-0407.70225 (PMC13076060; doi:10.1111/1753-0407.70225)

**Figure S1.** Manhattan Plot of GWAS Results for CKMs from MAGMA Analysis


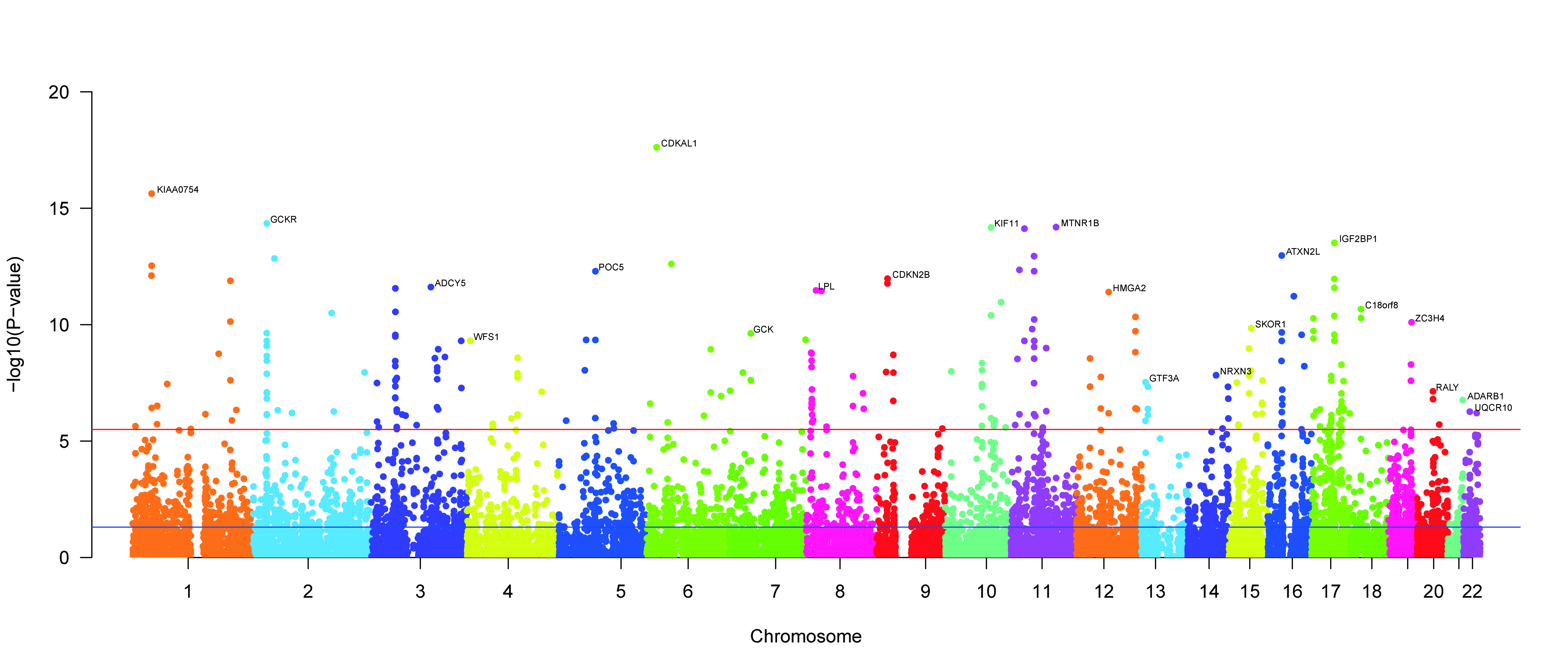

Supplement: Supplementary file 1 — Figure S1: Manhattan plot of GWAS results for CKMs from MAGMA analysis. [file JDB-18-e70225-s001.docx]
