# Supplementary material for: Genomic Structural Equation Modeling Reveals Cardiovascular‐Kidney‐Metabolic Syndrome Genetic Architecture
Source: J Diabetes. 2026 Apr 13;18(4):e70225. doi: 10.1111/1753-0407.70225 (PMC13076060; doi:10.1111/1753-0407.70225)
Supplement: Supplementary file 2 — Table S1: GWAS summary sources. Table S2: SNP heritability of genomic‐SEM phenotypes. Table S3: Model fit indices for the genomic structural equation model. Table S4: Factor loadings and residual covariances in genomic structural equation model of CKMs. Table S5: Novel SNP variants identified by genomic‐SEM. Table S6: Lead SNP identified by genomic‐SEM. Table S7: Risk locus identified by genomic‐SEM. Table S8: MAGMA risk gene annotation using genomic‐SEM. Table S9: Enriched pathways by MsigDB. Table S10: Enriched cell types in GWAS for CKMs. Table S11: Heritability enrichment across genomic functional and regulatory regions. Table S12: Polygenic risk score and genetic contribution across chromosomal regions. Table S13: Estimates of sample overlap and cryptic relatedness across the six source GWAS traits. [file JDB-18-e70225-s002.pdf]

Table S1. GWAS summary sources

**Download link**
https://gwas.mrcieu.ac.uk/datasets/ebi-a-GCST006368/
https://gwas.mrcieu.ac.uk/datasets/ebi-a-GCST000568/
https://gwas.mrcieu.ac.uk/datasets/ebi-a-GCST003374/
https://gwas.mrcieu.ac.uk/datasets/ebi-a-GCST90000615/
https://gwas.mrcieu.ac.uk/datasets/ebi-a-GCST003116/
https://gwas.mrcieu.ac.uk/datasets/ebi-a-GCST006867/

For Review Only

Table S2. SNP heritability of Genor

**Phenotype**	**NSNPs**	**h^2 (se)**	**λGC**
Type 2 diabetes	999241	0.4336 (0.0222)	1.4592
Coronary artery disease	1175954	0.2825 (0.0192)	1.0477
Serum 25-Hydroxyvitamin D levels	1124893	0.0851 (0.0091)	1.478
Body mass index	1161843	0.1641 (0.0058)	1.4921
Chronic kidney disease	977262	0.0176 (0.0057)	1.0475
Fasting glucose	1045689	0.0842 (0.0132)	1.0669

For Review Only

mic-SEM phenotypes

Mean ChiSquare	Intercept se	Ratio se
1.6389	1.0594 (0.017)	0.093 (0.0265)
1.1207	0.8857 (0.0082)	-0.9474 (0.0679)
1.7832	1.0522 (0.0238)	0.0667 (0.0304)
1.7897	0.7928 (0.0117)	-0.2623 (0.0148)
1.0636	1.0194 (0.0087)	0.3052 (0.137)
1.0724	0.9961 (0.0068)	-0.0545 (0.0939)

For Review Only

Table S3. Model Fit Indices for the Genomic Structural Equation Model

chisq	df	p_chisq	AIC	CFI	SRMR
9.03	9	0.43	33.03	0.99	0.14

For Review Only

Table S4. Factor Loadings and

lhs	op	rhs
F1	=~	Type 2 diabetes
F1	=~	Coronary artery disease
F1	=~	Serum 25-Hydroxyvitamin D levels
F1	=~	Body mass index
F1	=~	Chronic kidney disease
F1	=~	Fasting glucose
Type 2 diabetes	~~	Type 2 diabetes
Coronary artery disease	~~	Coronary artery disease
Serum 25-Hydroxyvitamin D levels	~~	Serum 25-Hydroxyvitamin D levels
Body mass index	~~	Body mass index
Chronic kidney disease	~~	Chronic kidney disease
Fasting glucose	~~	Fasting glucose

For Review Only

Residual Covariances in Genomic Structural Equation Model of CKMs

Unstandardized_Estimate	Unstandardized_SE	Standardized_Est	Standardized_SE
2.250748409	0.157588321	0.902885237	0.063216385
1.056855088	0.087911227	0.385518987	0.032068203
0.027680398	0.007023501	0.094896702	0.024078601
0.243638555	0.015292448	0.60148005	0.037753066
0.050289162	0.012686098	0.231068603	0.058289976
0.139733902	0.012469566	0.481437447	0.042962512
1.148382834	0.668361439	0.184798236	0.107553028
6.398238863	0.538582799	0.851374856	0.071665964
0.084317151	0.008992726	0.990994094	0.105693056
0.104717929	0.007779908	0.638221868	0.047416
0.044837101	0.015225995	0.946606508	0.321452784
0.064715343	0.013403628	0.768217826	0.159110567

For Review Only

p_value
2.81E-46
2.73E-33
8.11E-05
3.80E-57
7.37E-05
3.81E-29
0.085758993
1.51E-32
6.84E-21
2.69E-41
0.003231914
1.38E-06

For Review Only

Table S5. Novel SNP Variants Identified l

SNP	CHR	BP	other_allele	effect_allele	beta
rs2072948	1	6679848	T	C	-0.021956952
rs2282456	1	118169463	A	G	-0.02426242
rs227199	1	210265384	C	G	0.02312527
rs243034	2	60602892	T	C	0.025917166
rs2455830	3	15749213	T	C	0.020759116
rs12053904	3	53025837	T	C	0.025297878
rs938243	3	131559574	A	G	-0.034920719
rs6799056	3	135802350	T	C	-0.02705515
rs699165	3	136224697	G	A	-0.028276835
rs4678260	3	138089513	T	C	0.024494039
rs7693233	4	91254945	G	A	-0.02353642
rs1296328	4	137083193	C	A	0.024185254
rs13168288	5	106385815	G	A	0.030367432
rs12055786	6	153431125	T	C	-0.024943453
rs10276536	7	2086960	T	C	0.028423542
rs876954	8	8310923	A	G	-0.024475373
rs613080	8	9879451	G	C	0.031901948
rs4841662	8	11843758	A	G	-0.022768592
rs3808434	8	116559435	G	A	0.021645006
rs1154673	9	19052320	G	T	-0.025752538
rs11607855	11	47480403	T	C	-0.051982791
rs7102641	11	49553859	C	T	-0.040629461
rs3814707	11	65560785	A	G	0.025555455
rs2754087	14	25889339	A	T	-0.022598394
rs2010281	14	103862322	A	G	0.0241928
rs12593201	15	38844106	A	G	-0.025386344
rs1031664	15	51754897	A	C	0.020487323
rs9674995	17	9773043	A	C	-0.024078015
rs8072225	17	65912960	G	A	-0.028929138
rs7243357	18	56883319	G	T	0.028419965
rs12463359	19	46304585	T	G	0.02266167
rs9637192	21	46637413	C	T	-0.023741658

For Review Only

by Genomic-SEM

se	pval	N	
0.003914243	2.03E-08	123014.4375	7. 692696547
0.004365065	2.72E-08	123014.4375	7. 564814507
0.004197716	3.61E-08	123014.4375	7. 442671298
0.004098397	2.55E-10	123014.4375	9. 592914564
0.003799299	4.66E-08	123014.4375	7. 331894733
0.004267747	3.07E-09	123014.4375	8. 512539308
0.006345174	3.72E-08	123014.4375	7. 42909846
0.004497818	1.80E-09	123014.4375	8. 745462105
0.004643364	1.13E-09	123014.4375	8. 94653389
0.0038523	2.04E-10	123014.4375	9. 690343007
0.004042348	5.80E-09	123014.4375	8. 236668987
0.004114328	4.15E-09	123014.4375	8. 382472614
0.004934531	7.55E-10	123014.4375	9. 121932292
0.003876152	1.23E-10	123014.4375	9. 908731514
0.004612782	7.19E-10	123014.4375	9. 143433827
0.004029073	1.24E-09	123014.4375	8. 905786411
0.005642938	1.57E-08	123014.4375	7. 803330292
0.003945126	7.87E-09	123014.4375	8. 104291715
0.00373967	7.13E-09	123014.4375	8. 147199276
0.004141713	5.04E-10	123014.4375	9. 297552105
0.008426847	6.88E-10	123014.4375	9. 162104369
0.007088026	9.92E-09	123014.4375	8. 003578908
0.004420566	7.42E-09	123014.4375	8. 129353459
0.003956731	1.12E-08	123014.4375	7. 950533638
0.003905857	5.87E-10	123014.4375	9. 231630239
0.004613235	3.74E-08	123014.4375	7. 427691949
0.003757464	4.97E-08	123014.4375	7. 303772767
0.004408844	4.73E-08	123014.4375	7. 32544881
0.004958639	5.41E-09	123014.4375	8. 266918494
0.004892691	6.30E-09	123014.4375	8. 200825436
0.003853573	4.09E-09	123014.4375	8. 388744192
0.004125643	8.68E-09	123014.4375	8. 061387356

For Review Only

1.301029996

For Review Only

Table S(

Lead SNP	chr	pos	p
rs2072948	1	6679848	2.03E-08
rs2296172	1	39835817	2.13E-17
rs12088739	1	51506886	4.22E-09
rs2481665	1	62594677	2.41E-08
rs2613503	1	72839774	2.54E-19
rs197374	1	112289983	3.99E-08
rs2282456	1	118169463	2.72E-08
rs10913469	1	177913519	8.33E-16
rs2820315	1	201872264	5.40E-18
rs227199	1	210265384	3.61E-08
rs340874	1	214159256	1.52E-14
rs2820426	1	219660535	4.59E-08
rs13411762	2	554109	1.27E-08
rs13397165	2	653354	3.98E-23
rs10929925	2	6155557	4.49E-09
rs11676272	2	25141538	1.06E-13
rs780094	2	27741237	1.24E-25
rs7607777	2	43629931	4.97E-20
rs6545714	2	59307725	7.54E-12
rs243019	2	60585806	1.21E-14
rs243034	2	60602892	2.55E-10
rs1009358	2	65276452	1.45E-09
rs840967	2	65701757	4.88E-08
rs12617659	2	121309759	6.11E-09
rs7572970	2	161136656	7.59E-15
rs13389219	2	165528876	3.51E-16
rs9630985	2	181607676	1.85E-11
rs2943640	2	227093585	5.16E-26
rs7561798	2	228973660	4.79E-11
rs1801282	3	12393125	7.24E-15
rs2455830	3	15749213	4.66E-08
rs1496653	3	23454790	9.04E-12
rs2681781	3	49898273	8.75E-15
rs12053904	3	53025837	3.07E-09
rs11708067	3	123065778	1.99E-21
rs938243	3	131559574	3.72E-08
rs9856151	3	131574485	4.01E-09
rs6799056	3	135802350	1.80E-09
rs699165	3	136224697	1.13E-09
rs4678260	3	138089513	2.04E-10

For Review Only

rs9844972	3	150097635	1.10E-09
rs4472028	3	152053250	8.34E-11
rs7633675	3	185510613	6.02E-45
rs7647305	3	185834290	2.49E-15
rs6808574	3	187740523	4.55E-12
rs1801214	4	6303022	1.92E-32
rs10938397	4	45182527	1.86E-17
rs993380	4	83584496	2.55E-08
rs7693233	4	91254945	5.80E-09
rs151412	4	103138621	1.27E-09
rs7674212	4	103988899	4.84E-10
rs1296328	4	137083193	4.15E-09
rs7685296	4	153254121	4.52E-09
rs735949	4	185716232	4.26E-08
rs1061813	5	14847331	1.45E-08
rs1541681	5	53304620	1.48E-08
rs459193	5	55806751	1.91E-14
rs3936511	5	55860781	1.37E-15
rs2112347	5	75015242	1.32E-16
rs6878122	5	76427311	1.37E-11
rs1501672	5	87963761	7.17E-09
rs7729395	5	102100576	1.99E-13
rs13168288	5	106385815	7.55E-10
rs17672692	6	7214499	1.41E-09
rs1050226	6	7281654	1.66E-09
rs6903706	6	20518450	7.10E-10
rs7756992	6	20679709	6.22E-50
rs7738382	6	20746236	1.03E-08
rs4077404	6	20876613	5.24E-11
rs12173741	6	40394175	2.23E-09
rs987237	6	50803050	3.07E-27
rs2817419	6	50812906	5.48E-11
rs2246012	6	131898208	3.95E-11
rs12055786	6	153431125	1.23E-10
rs10276536	7	2086960	7.19E-10
rs17168486	7	14898282	5.28E-14
rs4719433	7	15065003	2.26E-17
rs849135	7	28196413	6.97E-32
rs215607	7	32338337	1.49E-09
rs2971669	7	44231778	8.51E-11
rs2299383	7	103418846	1.17E-10
rs10252771	7	117496890	6.03E-10

For Review Only

rs13234269	7	130429186	7.91E-11
rs2301916	7	156974127	1.79E-10
rs876954	8	8310923	1.24E-09
rs613080	8	9879451	1.57E-08
rs10100265	8	10633159	1.88E-13
rs4841662	8	11843758	7.87E-09
rs17411031	8	19852310	2.62E-11
rs11775287	8	30864339	2.45E-11
rs12681990	8	36859186	4.94E-10
rs6989203	8	41523745	2.39E-17
rs2060604	8	76650334	2.90E-10
rs7845219	8	95937502	1.65E-10
rs3808434	8	116559435	7.13E-09
rs3802177	8	118185025	3.25E-42
rs10974438	9	4291928	1.77E-12
rs1154673	9	19052320	5.04E-10
rs3731239	9	21974218	1.31E-10
rs4977574	9	22098574	6.12E-43
rs10811661	9	22134094	5.52E-46
rs16912921	9	28413461	5.00E-14
rs216368	9	33819955	1.09E-09
rs17791513	9	81905590	2.07E-09
rs2796441	9	84308948	6.49E-17
rs10114341	9	96919182	3.31E-08
rs11257655	10	12307894	4.51E-14
rs2633310	10	75594050	1.62E-10
rs753270	10	80964975	2.20E-09
rs10882028	10	93943900	4.76E-10
rs1111875	10	94462882	1.99E-32
rs11591741	10	101976501	4.03E-08
rs1547191	10	114612972	9.54E-10
rs7903146	10	114758349	1.00E-200
rs7896811	10	114766717	2.68E-29
rs10885414	10	114861304	4.75E-21
rs290483	10	114915214	3.44E-13
rs2421016	10	124167512	1.29E-08
rs6486120	11	13324142	7.61E-09
rs5215	11	17408630	4.47E-13
rs1519480	11	27675712	6.59E-20
rs4755726	11	43642130	1.73E-11
rs11607855	11	47480403	6.88E-10
rs11039266	11	47532395	8.55E-14

For Review Only

rs7102641	11	49553859	9.92E-09
rs3814707	11	65560785	7.42E-09
rs1552224	11	72433098	1.29E-16
rs10830963	11	92708710	7.36E-38
rs7932966	11	92792866	2.19E-12
rs10842994	12	27965150	2.04E-13
rs7138803	12	50247468	2.51E-15
rs2261181	12	66212318	1.99E-13
rs1042725	12	66358347	1.23E-11
rs1819844	12	68205604	1.39E-10
rs1169288	12	121416650	1.15E-14
rs825461	12	124561803	1.31E-09
rs9595630	13	33065443	4.23E-11
rs12429545	13	54102206	1.42E-12
rs9563576	13	58670147	3.44E-08
rs9540493	13	66205704	2.05E-08
rs1359790	13	80717156	1.07E-18
rs2754087	14	25889339	1.12E-08
rs10132280	14	25928179	6.99E-12
rs17522122	14	33302882	7.56E-11
rs7144011	14	79940383	1.49E-16
rs2015407	14	103274547	1.26E-09
rs2010281	14	103862322	5.87E-10
rs12593201	15	38844106	3.74E-08
rs1031664	15	51754897	4.97E-08
rs4502156	15	62383155	5.42E-09
rs11071759	15	63922474	3.35E-10
rs4776970	15	68080886	3.77E-13
rs7177055	15	77832762	1.36E-14
rs4932143	15	90372067	2.05E-08
rs12910825	15	91511260	1.04E-09
rs4788099	16	28855727	3.65E-14
rs7203521	16	53769293	2.59E-21
rs1421085	16	53800954	2.00E-56
rs17218700	16	53844579	5.70E-10
rs6499653	16	53877592	3.16E-11
rs244415	16	69666683	5.72E-14
rs889512	16	75242012	2.03E-16
rs2925979	16	81534790	8.97E-11
rs8068804	17	3985864	2.79E-15
rs9674995	17	9773043	4.73E-08
rs12945601	17	17653411	1.59E-08
rs12600570	17	40261545	2.18E-08

For Review Only

rs6963	17	40731597	3.14E-10
rs4794018	17	47093398	9.11E-18
rs302864	17	56757584	1.85E-08
rs17631783	17	61687600	1.23E-10
rs8072225	17	65912960	5.41E-09
rs1788785	18	21142340	5.26E-13
rs7243357	18	56883319	6.30E-09
rs12970134	18	57884750	1.39E-27
rs17066856	18	58049656	3.28E-08
rs8108269	19	46158513	1.16E-12
rs12463359	19	46304585	4.09E-09
rs2303108	19	47589895	8.40E-14
rs6059662	20	32675727	2.53E-09
rs6066149	20	45602638	4.58E-08
rs9637192	21	46637413	8.68E-09
rs7290175	22	30598097	2.81E-08

For Review Only

6. Lead SNP Identified by Genomic-SEM

**Independent SNPs**
rs2072948
rs2296172;rs3738676;rs17513135;rs12028034;rs636083
rs12088739
rs2481665
rs2613503;rs7530496;rs1545933;rs2126077;rs990871
rs197374
rs2282456
rs10913469;rs12737338;rs576101;rs580294;rs591120
rs2820315;rs504988;rs2678209;rs2820292;rs1022361;rs10920259
rs227199
rs340874;rs6698217;rs7529073
rs2820426
rs13411762;rs13029479
rs13397165;rs10188118;rs13029479;rs2903495
rs10929925
rs11676272;rs6726261
rs780094;rs13002853;rs12104449;rs780110
rs7607777;rs17031133;rs1367173;rs11899984
rs6545714
rs243019;rs243015;rs181680;rs12464462;rs184838
rs243034;rs184839;rs12464462;rs243015;rs181680
rs1009358
rs840967
rs12617659
rs7572970;rs12692596;rs6742799
rs13389219;rs7607980;rs355838
rs9630985;rs1528435
rs2943640;rs2673129;rs10194882;rs1522803;rs4645008
rs7561798;rs4283409;rs10209496
rs1801282;rs7621569;rs7629805
rs2455830
rs1496653
rs2681781;rs3774734;rs6446298
rs12053904;rs2239547
rs11708067;rs6784930
rs938243
rs9856151;rs876424;rs1320903
rs6799056
rs699165;rs610860
rs4678260

For Review Only

rs9844972
rs4472028;rs3796273
rs7633675;rs1374910;rs11705701;rs10513800
rs7647305;rs4234589
rs6808574
rs1801214;rs4234733;rs4234726
rs10938397;rs1849338;rs16858082
rs993380
rs7693233;rs1037926
rs151412
rs7674212;rs13112128;rs223420;rs10516497
rs1296328
rs7685296
rs735949
rs1061813
rs1541681
rs459193
rs3936511
rs2112347;rs4704235;rs253409;rs34341;rs1817869
rs6878122;rs7707527
rs1501672
rs7729395;rs13188193
rs13168288
rs17672692
rs1050226
rs6903706;rs9366354;rs1997777;rs7755830;rs7775523
rs7756992;rs1040558;rs12110493;rs6928012;rs7741604;rs9350276;rs6909558;rs9295482;rs471254
0;rs9465890;rs9350294;rs7775523;rs6904566;rs9366354;rs1997777;rs7768642;rs7755830;rs16884
rs7738382;rs6909558;rs4712540;rs9465890;rs7775523;rs7768642;rs9356743;rs12527373;rs104055
rs4077404;rs7775523;rs9366354;rs1997777;rs7755830;rs1012635;rs9350276;rs9295482;rs4712540
rs12173741
rs987237;rs9473932;rs4082052;rs17540418;rs12528998
rs2817419;rs9473932;rs9395635
rs2246012
rs12055786;rs4385321
rs10276536;rs1403175
rs17168486;rs17168465
rs4719433;rs17168570;rs1005256
rs849135;rs530649;rs508347;rs849327;rs10276070;rs917115;rs849139;rs849140
rs215607
rs2971669
rs2299383;rs7341475
rs10252771

For Review Only

rs13234269
rs2301916;rs1182397
rs876954;rs13268432;rs2409096;rs2010390;rs6990912;rs2945238
rs613080;rs2062332;rs7832708;rs4841407;rs6985109;rs11786541;rs609792
rs10100265;rs2409676;rs6985109;rs2409742;rs2572450;rs2618451;rs7822958;rs11777273;rs11786541;rs609792;rs2062332;rs1484641;rs7832708;rs4841407;rs919555
rs4841662;rs2409742;rs2572450;rs2618451;rs7822958;rs11777273
rs17411031;rs894210;rs6586891;rs264;rs17482753
rs11775287;rs7827210;rs9297164;rs2543622
rs12681990
rs6989203;rs12549902
rs2060604;rs12547392
rs7845219
rs3808434
rs3802177;rs2466293;rs2466291;rs2464573;rs7005140
rs10974438
rs1154673
rs3731239;rs3218009;rs3217992;rs10757264;rs1412829;rs8181047
rs4977574;rs3218009;rs3217992;rs10757264;rs1412829;rs8181047
rs10811661;rs7020996
rs16912921;rs10757739
rs216368
rs17791513
rs2796441;rs2129107
rs10114341
rs11257655;rs7069060
rs2633310;rs11000746
rs753270
rs10882028;rs11187094
rs1111875;rs2488075;rs2798253;rs17445328;rs11187094;rs6583833
rs11591741
rs1547191
rs7903146;rs10885402;rs12266632;rs11196212;rs7901275;rs10128255
rs7896811;rs11196181;rs10885402
rs10885414;rs4917646;rs4081699
rs290483;rs4081699;rs4917646
rs2421016
rs6486120
rs5215
rs1519480;rs10767664;rs12273363;rs4514364;rs10835197
rs4755726;rs2176598;rs2625387
rs11607855
rs11039266;rs12226431;rs7124681

For Review Only

rs7102641
rs3814707
rs1552224;rs1783598;rs12575364
rs10830963;rs12222793;rs11020107;rs2166706;rs7951037
rs7932966;rs16918495
rs10842994
rs7138803;rs11836282;rs10875976;rs4898536
rs2261181;rs7134682
rs1042725
rs1819844
rs1169288;rs12427353;rs1169302;rs2708081;rs661647;rs4767941;rs11065360;rs7953249
rs825461
rs9595630
rs12429545
rs9563576
rs9540493
rs1359790;rs9601328;rs679805;rs537435
rs2754087
rs10132280
rs17522122
rs7144011;rs7141420
rs2015407;rs7143963
rs2010281
rs12593201
rs1031664
rs4502156
rs11071759;rs11635117
rs4776970;rs16951275
rs7177055;rs907374;rs12591335
rs4932143
rs12910825;rs12899811
rs4788099
rs7203521;rs7186521;rs13333228;rs2058908;rs4783819
rs1421085;rs2058908;rs4783819;rs7186521
rs17218700
rs6499653
rs244415;rs3790085;rs3790083;rs4985377;rs889400
rs889512;rs7202877;rs7190458;rs4261573;rs17764278
rs2925979
rs8068804;rs10521129;rs4790587;rs4508454;rs17763551
rs9674995
rs12945601
rs12600570;rs2062213

For Review Only

rs6963;rs2062213
rs4794018;rs953259;rs7217007;rs11650158;rs9894220;rs8078510;rs2411759
rs302864
rs17631783;rs9893518;rs11655589
rs8072225;rs12600551
rs1788785;rs1805082
rs7243357
rs12970134;rs9956279;rs1942863;rs11660783;rs6567157;rs1673518;rs619662
rs17066856
rs8108269
rs12463359;rs4803848
rs2303108
rs6059662
rs6066149
rs9637192
rs7290175

For Review Only

Table S7. Risk locus Identified by Genom

Sequence	Locus	SNP
1	1:6679848:C:T	rs2072948
2	1:39835817:A:G	rs2296172
3	1:51506886:A:G	rs12088739
4	1:62594677:C:T	rs2481665
5	1:72839774:A:C	rs2613503
6	1:112289983:C:T	rs197374
7	1:118169463:A:G	rs2282456
8	1:177913519:C:T	rs10913469
9	1:201872264:C:T	rs2820315
10	1:210265384:C:G	rs227199
11	1:214159256:C:T	rs340874
12	1:219660535:A:G	rs2820426
13	2:653354:A:G	rs13397165
14	2:6155557:A:C	rs10929925
15	2:25141538:A:G	rs11676272
16	2:27741237:C:T	rs780094
17	2:43629931:G:T	rs7607777
18	2:59307725:A:G	rs6545714
19	2:60585806:C:T	rs243019
20	2:65276452:C:T	rs1009358
21	2:65701757:A:C	rs840967
22	2:121309759:C:T	rs12617659
23	2:161136656:A:G	rs7572970
24	2:165528876:C:T	rs13389219
25	2:181607676:A:C	rs9630985
26	2:227093585:A:C	rs2943640
27	2:228973660:A:G	rs7561798
28	3:12393125:C:G	rs1801282
29	3:15749213:C:T	rs2455830
30	3:23454790:A:G	rs1496653
31	3:49898273:A:G	rs2681781
32	3:53025837:C:T	rs12053904
33	3:123065778:A:G	rs11708067
34	3:131574485:A:G	rs9856151
35	3:136224697:A:G	rs699165
36	3:138089513:C:T	rs4678260
37	3:150097635:C:G	rs9844972
38	3:152053250:C:T	rs4472028
39	3:185510613:G:T	rs7633675
40	3:187740523:C:T	rs6808574
41	4:6303022:C:T	rs1801214

For Review Only

42	4:45182527:A:G	rs10938397
43	4:83584496:A:G	rs993380
44	4:91254945:A:G	rs7693233
45	4:103138621:C:T	rs151412
46	4:103988899:G:T	rs7674212
47	4:137083193:A:C	rs1296328
48	4:153254121:C:T	rs7685296
49	4:185716232:C:T	rs735949
50	5:14847331:A:G	rs1061813
51	5:53304620:G:T	rs1541681
52	5:55860781:A:G	rs3936511
53	5:75015242:G:T	rs2112347
54	5:76427311:A:G	rs6878122
55	5:87963761:A:G	rs1501672
56	5:102100576:C:T	rs7729395
57	5:106385815:A:G	rs13168288
58	6:7214499:C:T	rs17672692
59	6:20679709:A:G	rs7756992
60	6:40394175:C:T	rs12173741
61	6:50803050:A:G	rs987237
62	6:131898208:C:T	rs2246012
63	6:153431125:C:T	rs12055786
64	7:2086960:C:T	rs10276536
65	7:15065003:C:T	rs4719433
66	7:28196413:A:G	rs849135
67	7:32338337:A:G	rs215607
68	7:44231778:C:T	rs2971669
69	7:103418846:C:T	rs2299383
70	7:117496890:G:T	rs10252771
71	7:130429186:A:T	rs13234269
72	7:156974127:G:T	rs2301916
73	8:8310923:A:G	rs876954
74	8:10633159:A:C	rs10100265
75	8:19852310:C:G	rs17411031
76	8:30864339:C:T	rs11775287
77	8:36859186:C:T	rs12681990
78	8:41523745:A:G	rs6989203
79	8:76650334:C:T	rs2060604
80	8:95937502:C:T	rs7845219
81	8:116559435:A:G	rs3808434
82	8:118185025:A:G	rs3802177
83	9:4291928:A:C	rs10974438
84	9:19052320:G:T	rs1154673

For Review Only

85	9:22134094:C:T	rs10811661
86	9:28413461:A:C	rs16912921
87	9:33819955:A:G	rs216368
88	9:81905590:A:G	rs17791513
89	9:84308948:A:G	rs2796441
90	9:96919182:C:T	rs10114341
91	10:12307894:C:T	rs11257655
92	10:75594050:G:T	rs2633310
93	10:80964975:C:T	rs753270
94	10:94462882:C:T	rs1111875
95	10:101976501:C:G	rs11591741
96	10:114758349:C:T	rs7903146
97	10:124167512:C:T	rs2421016
98	11:13324142:G:T	rs6486120
99	11:17408630:C:T	rs5215
100	11:27675712:C:T	rs1519480
101	11:43642130:G:T	rs4755726
102	11:47532395:G:T	rs11039266
103	11:65560785:A:G	rs3814707
104	11:72433098:A:C	rs1552224
105	11:92708710:C:G	rs10830963
106	12:27965150:C:T	rs10842994
107	12:50247468:A:G	rs7138803
108	12:66212318:C:T	rs2261181
109	12:68205604:A:G	rs1819844
110	12:121416650:A:C	rs1169288
111	12:124561803:C:T	rs825461
112	13:33065443:G:T	rs9595630
113	13:54102206:A:G	rs12429545
114	13:58670147:C:T	rs9563576
115	13:66205704:A:G	rs9540493
116	13:80717156:A:G	rs1359790
117	14:25928179:A:C	rs10132280
118	14:33302882:G:T	rs17522122
119	14:79940383:G:T	rs7144011
120	14:103274547:A:G	rs2015407
121	14:103862322:A:G	rs2010281
122	15:38844106:A:G	rs12593201
123	15:51754897:A:C	rs1031664
124	15:62383155:C:T	rs4502156
125	15:63922474:C:T	rs11071759
126	15:68080886:A:T	rs4776970
127	15:77832762:A:G	rs7177055

For Review Only

128	15:90372067:C:G	rs4932143
129	15:91511260:A:G	rs12910825
130	16:28855727:A:G	rs4788099
131	16:53800954:C:T	rs1421085
132	16:69666683:A:G	rs244415
133	16:75242012:C:G	rs889512
134	16:81534790:C:T	rs2925979
135	17:3985864:A:G	rs8068804
136	17:9773043:A:C	rs9674995
137	17:17653411:C:T	rs12945601
138	17:40731597:A:T	rs6963
139	17:47093398:C:T	rs4794018
140	17:56757584:A:G	rs302864
141	17:61687600:C:T	rs17631783
142	17:65912960:A:G	rs8072225
143	18:21142340:C:T	rs1788785
144	18:56883319:G:T	rs7243357
145	18:57884750:A:G	rs12970134
146	19:46158513:G:T	rs8108269
147	19:47589895:C:T	rs2303108
148	20:32675727:A:G	rs6059662
149	20:45602638:A:G	rs6066149
150	21:46637413:C:T	rs9637192
151	22:30598097:A:T	rs7290175

For Review Only

ıic-SEM

chr	pos	p
1	6679848	2.03E-08
1	39835817	2.13E-17
1	51506886	4.22E-09
1	62594677	2.41E-08
1	72839774	2.54E-19
1	112289983	3.99E-08
1	118169463	2.72E-08
1	177913519	8.33E-16
1	201872264	5.40E-18
1	210265384	3.61E-08
1	214159256	1.52E-14
1	219660535	4.59E-08
2	653354	3.98E-23
2	6155557	4.49E-09
2	25141538	1.06E-13
2	27741237	1.24E-25
2	43629931	4.97E-20
2	59307725	7.54E-12
2	60585806	1.21E-14
2	65276452	1.45E-09
2	65701757	4.88E-08
2	121309759	6.11E-09
2	161136656	7.59E-15
2	165528876	3.51E-16
2	181607676	1.85E-11
2	227093585	5.16E-26
2	228973660	4.79E-11
3	12393125	7.24E-15
3	15749213	4.66E-08
3	23454790	9.04E-12
3	49898273	8.75E-15
3	53025837	3.07E-09
3	123065778	1.99E-21
3	131574485	4.01E-09
3	136224697	1.13E-09
3	138089513	2.04E-10
3	150097635	1.10E-09
3	152053250	8.34E-11
3	185510613	6.02E-45
3	187740523	4.55E-12
4	6303022	1.92E-32

For Review Only

4	45182527	1.86E-17
4	83584496	2.55E-08
4	91254945	5.80E-09
4	103138621	1.27E-09
4	103988899	4.84E-10
4	137083193	4.15E-09
4	153254121	4.52E-09
4	185716232	4.26E-08
5	14847331	1.45E-08
5	53304620	1.48E-08
5	55860781	1.37E-15
5	75015242	1.32E-16
5	76427311	1.37E-11
5	87963761	7.17E-09
5	102100576	1.99E-13
5	106385815	7.55E-10
6	7214499	1.41E-09
6	20679709	6.22E-50
6	40394175	2.23E-09
6	50803050	3.07E-27
6	131898208	3.95E-11
6	153431125	1.23E-10
7	2086960	7.19E-10
7	15065003	2.26E-17
7	28196413	6.97E-32
7	32338337	1.49E-09
7	44231778	8.51E-11
7	103418846	1.17E-10
7	117496890	6.03E-10
7	130429186	7.91E-11
7	156974127	1.79E-10
8	8310923	1.24E-09
8	10633159	1.88E-13
8	19852310	2.62E-11
8	30864339	2.45E-11
8	36859186	4.94E-10
8	41523745	2.39E-17
8	76650334	2.90E-10
8	95937502	1.65E-10
8	116559435	7.13E-09
8	118185025	3.25E-42
9	4291928	1.77E-12
9	19052320	5.04E-10

For Review Only

9	22134094	5.52E-46
9	28413461	5.00E-14
9	33819955	1.09E-09
9	81905590	2.07E-09
9	84308948	6.49E-17
9	96919182	3.31E-08
10	12307894	4.51E-14
10	75594050	1.62E-10
10	80964975	2.20E-09
10	94462882	1.99E-32
10	101976501	4.03E-08
10	114758349	1.00E-200
10	124167512	1.29E-08
11	13324142	7.61E-09
11	17408630	4.47E-13
11	27675712	6.59E-20
11	43642130	1.73E-11
11	47532395	8.55E-14
11	65560785	7.42E-09
11	72433098	1.29E-16
11	92708710	7.36E-38
12	27965150	2.04E-13
12	50247468	2.51E-15
12	66212318	1.99E-13
12	68205604	1.39E-10
12	121416650	1.15E-14
12	124561803	1.31E-09
13	33065443	4.23E-11
13	54102206	1.42E-12
13	58670147	3.44E-08
13	66205704	2.05E-08
13	80717156	1.07E-18
14	25928179	6.99E-12
14	33302882	7.56E-11
14	79940383	1.49E-16
14	103274547	1.26E-09
14	103862322	5.87E-10
15	38844106	3.74E-08
15	51754897	4.97E-08
15	62383155	5.42E-09
15	63922474	3.35E-10
15	68080886	3.77E-13
15	77832762	1.36E-14

For Review Only

15	90372067	2.05E-08
15	91511260	1.04E-09
16	28855727	3.65E-14
16	53800954	2.00E-56
16	69666683	5.72E-14
16	75242012	2.03E-16
16	81534790	8.97E-11
17	3985864	2.79E-15
17	9773043	4.73E-08
17	17653411	1.59E-08
17	40731597	3.14E-10
17	47093398	9.11E-18
17	56757584	1.85E-08
17	61687600	1.23E-10
17	65912960	5.41E-09
18	21142340	5.26E-13
18	56883319	6.30E-09
18	57884750	1.39E-27
19	46158513	1.16E-12
19	47589895	8.40E-14
20	32675727	2.53E-09
20	45602638	4.58E-08
21	46637413	8.68E-09
22	30598097	2.81E-08

For Review Only

Table S8. MAGMA Risk Gene Annotation Using Gen

Gene	chr	start	end	strand	type
LPL	8	19759228	19824769	1	protein_coding
ANKDD1B	5	74907284	74967671	1	protein_coding
POC5	5	74969949	75013313	-1	protein_coding
MTCH2	11	47638867	47664175	-1	protein_coding
THADA	2	43393800	43823185	-1	protein_coding
HNF1A	12	121416346	121440315	1	protein_coding
UBAP2	9	33921691	34048947	-1	protein_coding
BDNF	11	27676440	27743605	-1	protein_coding
C18orf8	18	21083473	21111746	1	protein_coding
NPC1	18	21086148	21166862	-1	protein_coding
SH2B1	16	28857921	28885526	1	protein_coding
WFS1	4	6271576	6304992	1	protein_coding
ATP5G1	17	46970127	46973233	1	protein_coding
IPO9	1	201798269	201853422	1	protein_coding
LMOD1	1	201865580	201915715	-1	protein_coding
SHISA4	1	201857808	201861434	1	protein_coding
MLX	17	40719086	40725257	1	protein_coding
PINX1	8	10622473	10697394	-1	protein_coding
PRC1	15	91509270	91538859	-1	protein_coding
SOX7	8	10581278	10697357	-1	protein_coding
SKOR1	15	68112042	68126899	1	protein_coding
SLC39A13	11	47428683	47438047	1	protein_coding
TCF7L2	10	114710009	114927437	1	protein_coding
WWP2	16	69796209	69975644	1	protein_coding
FTO	16	53737875	54155853	1	protein_coding
CDKAL1	6	20534688	21232635	1	protein_coding
ZC3H4	19	47567444	47617009	-1	protein_coding
CELF1	11	47487496	47587121	-1	protein_coding
KBTBD4	11	47593749	47600567	-1	protein_coding
MAP2K5	15	67835047	68099461	1	protein_coding
NDUFS3	11	47586888	47606114	1	protein_coding
PABPC4	1	40026488	40042462	-1	protein_coding
ARNTL	11	13298199	13408813	1	protein_coding
JAZF1	7	27870192	28220362	-1	protein_coding
IGF2BP1	17	47074774	47133012	1	protein_coding
TFAP2B	6	50786436	50815326	1	protein_coding
MACF1	1	39546988	39952849	1	protein_coding
AC022431.2	5	55807394	55902059	-1	protein_coding
MED23	6	131895106	131949369	-1	protein_coding
FAIM2	12	50260679	50298000	-1	protein_coding
SNF8	17	47006678	47022479	-1	protein_coding

For Review Only

UBE2Z	17	46985731	47006418	1	protein_coding
ADCY5	3	123001143	123168605	-1	protein_coding
MSL2	3	135867764	135916083	-1	protein_coding
UBE2D3	4	103715540	103790053	-1	protein_coding
RBMS1	2	161128662	161350305	-1	protein_coding
ARL15	5	53179775	53606412	-1	protein_coding
GTF3C2	2	27548716	27579868	-1	protein_coding
ATP2A1	16	28889726	28915830	1	protein_coding
CAMKV	3	49895421	49907655	-1	protein_coding
SPPL3	12	121200313	121342174	-1	protein_coding
EIF2B4	2	27587219	27593353	-1	protein_coding
RBM5	3	50126341	50156454	1	protein_coding
SNX17	2	27593389	27599995	1	protein_coding
ARAP1	11	72396114	72504644	-1	protein_coding
NRXN3	14	78708734	80330762	1	protein_coding
MAP3K3	17	61699775	61773663	1	protein_coding
NDST2	10	75561669	75571589	-1	protein_coding
RP11-574K11.31	10	75556272	75571409	-1	protein_coding
IP6K1	3	49761727	49823975	-1	protein_coding
NEGR1	1	71861623	72748417	-1	protein_coding
RBM6	3	49977440	50137478	1	protein_coding
TRAIP	3	49866034	49894007	-1	protein_coding
HERC1	15	63900817	64126141	-1	protein_coding
ZZEF1	17	3907739	4046314	-1	protein_coding
IGF2BP2	3	185361527	185542844	-1	protein_coding
HMGA2	12	66217911	66360075	1	protein_coding
HAUS6	9	19053141	19103117	-1	protein_coding
STAG1	3	136055077	136471220	-1	protein_coding
GIP	17	47035916	47045958	-1	protein_coding
GCK	7	44183872	44237769	-1	protein_coding
NDUFAF6	8	95907995	96128683	1	protein_coding
CPNE4	3	131252399	132004254	-1	protein_coding
NUP160	11	47799639	47870107	-1	protein_coding
CLN3	16	28477974	28503333	-1	protein_coding
HSD17B12	11	43577986	43878167	1	protein_coding
FNBP4	11	47738072	47788995	-1	protein_coding
BPTF	17	65821640	65980494	1	protein_coding
MBNL1	3	151961617	152183569	1	protein_coding
BCAR1	16	75262928	75301951	-1	protein_coding
MRAS	3	138066539	138124375	1	protein_coding
GCKR	2	27719709	27746554	1	protein_coding
IFT172	2	27667238	27712656	-1	protein_coding
UBE3C	7	156931607	157062066	1	protein_coding

For Review Only

SEC16B	1	177893091	177953438	-1	protein_coding
MSRA	8	9911778	10286401	1	protein_coding
PURG	8	30853321	30891231	-1	protein_coding
C2orf16	2	27799389	27805588	1	protein_coding
SEC24C	10	75504120	75531919	1	protein_coding
ATP6V0A1	17	40610862	40674629	1	protein_coding
KIAA0754	1	39876151	39882154	1	protein_coding
KIF11	10	94353043	94415150	1	protein_coding
SLC9B1	4	103806205	103940896	-1	protein_coding
AGBL2	11	47681143	47736941	-1	protein_coding
KCNJ11	11	17407406	17410878	-1	protein_coding
RSPH6A	19	46298968	46318577	-1	protein_coding
XKR6	8	10753555	11058875	-1	protein_coding
TIMP4	3	12194551	12200851	-1	protein_coding
SLC9B2	4	103941025	104006986	-1	protein_coding
KLHL42	12	27932953	27955973	1	protein_coding
MANSC4	12	27915671	27924209	-1	protein_coding
TRAF3	14	103243813	103377837	1	protein_coding
CISD2	4	103790135	103810399	1	protein_coding
CTD-2330K9.3	3	49941278	49954370	1	protein_coding
MON1A	3	49946302	49967606	-1	protein_coding
RP11-894J14.5	3	53003135	53133469	-1	protein_coding
SPHKAP	2	228844666	229046361	-1	protein_coding
TTLL6	17	46839597	46894576	-1	protein_coding
PPM1G	2	27604061	27632554	-1	protein_coding
RP11-145E5.5	9	21802635	22032985	1	protein_coding
ANKFY1	17	4067201	4167274	-1	protein_coding
CDKN2B	9	22002902	22009362	-1	protein_coding
CYB5D2	17	4046462	4090605	1	protein_coding
MTNR1B	11	92702886	92718232	1	protein_coding
N4BP2L2	13	33006554	33112970	-1	protein_coding
PCCB	3	135969148	136056738	1	protein_coding
TUFM	16	28853732	28857729	-1	protein_coding
UBE2R2	9	33817565	33920402	1	protein_coding

For Review Only

omic-SEM

GenomicLocus	Max CADD
75	50
53	45
53	45
102	25
17	23.5
110	23.4
87	23.4
100	22.5
143	22.5
143	22.5
130	22.5
41	22.2
139	22.1
9	22.1
9	22.1
9	22.1
138	22
74	22
129	22
74	22
126	21.9
102	21.7
96	21.7
132	21.6
131	21.4
59	21.3
147	21
102	20.9
102	20.9
126	20.9
102	20.9
2	20.9
98	20.4
66	20.2
139	19.98
61	19.87
2	19.78
52	19.72
62	19.68
107	19.54
139	19.46

For Review Only

139	19.46
33	19.37
35	19.3
46	19.24
23	19.11
51	19.1
16	18.95
130	18.68
31	18.51
110	18.48
16	18.47
31	18.47
16	18.47
104	18.45
119	18.29
141	18.21
92	17.98
92	17.98
31	17.96
5	17.85
31	17.77
31	17.61
125	17.56
135	17.19
39	17.08
108	16.75
84	16.65
35	16.64
139	16.46
68	16.3
80	16.24
34	16.22
102	16.15
130	16.07
101	16.07
102	16.01
142	15.96
38	15.96
133	15.88
36	15.58
16	15.52
16	15.52
72	15.48

For Review Only

8	15.31
74	15.16
76	15.16
16	14.88
92	14.8
138	14.68
2	14.68
94	14.57
46	14.57
102	14.46
99	14.43
146	14.08
74	14.04
28	13.82
46	13.65
106	13.6
106	13.6
120	13.53
46	13.18
31	13.15
31	13.15
32	13.12
27	12.92
139	12.87
16	12.85
85	12.84
135	12.47
85	0
135	0
105	0
112	0
35	0
130	0
87	0

For Review Only

Category
Curated_gene_sets
Curated_gene_sets
Canonical_Pathways
Canonical_Pathways
Canonical_Pathways
Canonical_Pathways
TF_targets
TF_targets
TF_targets
TF_targets
TF_targets
TF_targets
TF_targets
TF_targets
TF_targets
TF_targets
TF_targets
TF_targets
TF_targets
KEGG
GO_bp
GO_bp
GO_bp
GO_bp
GO_bp
GO_bp
GO_bp
GO_bp
GO_bp
GO_bp
GO_bp
GO_bp
GO_bp
GO_bp
GO_bp
GO_bp
GO_bp
GO_bp
GO_bp

For Review Only

GO_bp
Wikipathways
Wikipathways
GWAScatalog
GWAScatalog
GWAScatalog
GWAScatalog
GWAScatalog
GWAScatalog
GWAScatalog
GWAScatalog
GWAScatalog
GWAScatalog
GWAScatalog
GWAScatalog
GWAScatalog
GWAScatalog
GWAScatalog
GWAScatalog
GWAScatalog
GWAScatalog
GWAScatalog
GWAScatalog
GWAScatalog
GWAScatalog
GWAScatalog
GWAScatalog
GWAScatalog
GWAScatalog
GWAScatalog
GWAScatalog
GWAScatalog
GWAScatalog
GWAScatalog
GWAScatalog
GWAScatalog
GWAScatalog
GWAScatalog
GWAScatalog
GWAScatalog
GWAScatalog

For Review Only

GWAScatalog
GWAScatalog
GWAScatalog
GWAScatalog
GWAScatalog
GWAScatalog
GWAScatalog
GWAScatalog
GWAScatalog
GWAScatalog
GWAScatalog
GWAScatalog
GWAScatalog
GWAScatalog
GWAScatalog
GWAScatalog
GWAScatalog
GWAScatalog
GWAScatalog
GWAScatalog
GWAScatalog
GWAScatalog
GWAScatalog
GWAScatalog
GWAScatalog
GWAScatalog
GWAScatalog
GWAScatalog
GWAScatalog
GWAScatalog
GWAScatalog
GWAScatalog
GWAScatalog
GWAScatalog
GWAScatalog
GWAScatalog
GWAScatalog
GWAScatalog
GWAScatalog
GWAScatalog
GWAScatalog
GWAScatalog
GWAScatalog

For Review Only

GWAScatalog
GWAScatalog
GWAScatalog
GWAScatalog
GWAScatalog
GWAScatalog
GWAScatalog
GWAScatalog
GWAScatalog
GWAScatalog
GWAScatalog
GWAScatalog
GWAScatalog
GWAScatalog
GWAScatalog
GWAScatalog
GWAScatalog
GWAScatalog
GWAScatalog
GWAScatalog
GWAScatalog
GWAScatalog
GWAScatalog
GWAScatalog
GWAScatalog
GWAScatalog
GWAScatalog
GWAScatalog
GWAScatalog
GWAScatalog
GWAScatalog
GWAScatalog
GWAScatalog
GWAScatalog
GWAScatalog
GWAScatalog
GWAScatalog
GWAScatalog
GWAScatalog
GWAScatalog
GWAScatalog
GWAScatalog
GWAScatalog

For Review Only

GWAScatalog
GWAScatalog
GWAScatalog
GWAScatalog
GWAScatalog
GWAScatalog
GWAScatalog
GWAScatalog
GWAScatalog
GWAScatalog
GWAScatalog
GWAScatalog
GWAScatalog
GWAScatalog
GWAScatalog
GWAScatalog
GWAScatalog
GWAScatalog
GWAScatalog
GWAScatalog
GWAScatalog
GWAScatalog
GWAScatalog
GWAScatalog
GWAScatalog
GWAScatalog
GWAScatalog
GWAScatalog
GWAScatalog
GWAScatalog
GWAScatalog
GWAScatalog
GWAScatalog
GWAScatalog
GWAScatalog
GWAScatalog
GWAScatalog
GWAScatalog
GWAScatalog
GWAScatalog
GWAScatalog
GWAScatalog
GWAScatalog

For Review Only

GWAScatalog
GWAScatalog
GWAScatalog
GWAScatalog
GWAScatalog
GWAScatalog
GWAScatalog
GWAScatalog
GWAScatalog
GWAScatalog
GWAScatalog
GWAScatalog
GWAScatalog
GWAScatalog
GWAScatalog
GWAScatalog
GWAScatalog
GWAScatalog
GWAScatalog
GWAScatalog
GWAScatalog
GWAScatalog
GWAScatalog
GWAScatalog
GWAScatalog
GWAScatalog
GWAScatalog
GWAScatalog
GWAScatalog
GWAScatalog
GWAScatalog
GWAScatalog
GWAScatalog
GWAScatalog
GWAScatalog
GWAScatalog
GWAScatalog
GWAScatalog
GWAScatalog
GWAScatalog
GWAScatalog
GWAScatalog
GWAScatalog

For Review Only

GWAScatalog
GWAScatalog
GWAScatalog
GWAScatalog
GWAScatalog
GWAScatalog
GWAScatalog
GWAScatalog
GWAScatalog
GWAScatalog
GWAScatalog
GWAScatalog
GWAScatalog
GWAScatalog
GWAScatalog
GWAScatalog
GWAScatalog
GWAScatalog
GWAScatalog
GWAScatalog
GWAScatalog
GWAScatalog
GWAScatalog
GWAScatalog
GWAScatalog
GWAScatalog
GWAScatalog
GWAScatalog
GWAScatalog
GWAScatalog
GWAScatalog
GWAScatalog
GWAScatalog
GWAScatalog
GWAScatalog
GWAScatalog
GWAScatalog
GWAScatalog
GWAScatalog
GWAScatalog
GWAScatalog
GWAScatalog
GWAScatalog

For Review Only

GWAScatalog
GWAScatalog
GWAScatalog
GWAScatalog
GWAScatalog
GWAScatalog
GWAScatalog
GWAScatalog
GWAScatalog
GWAScatalog
GWAScatalog
GWAScatalog
GWAScatalog
Positional_gene_sets
Positional_gene_sets
Positional_gene_sets
Positional_gene_sets
Positional_gene_sets
Positional_gene_sets
Positional_gene_sets
Positional_gene_sets
Positional_gene_sets
BioCarta
Immunologic_signatures
Chemical_and_Genetic_pertubation
Chemical_and_Genetic_pertubation
Chemical_and_Genetic_pertubation

For Review Only

Table S9. Enriched Pat

**GeneSet**
DACOSTA_UV_RESPONSE_VIA_ERCC3_DN
NIKOLSKY_BREAST_CANCER_17Q21_Q25_AMPLICON
WP_16P112_PROXIMAL_DELETION_SYNDROME
WP_16P112_DISTAL_DELETION_SYNDROME
REACTOME_ANTIGEN_PROCESSING_UBIQUITINATION_PROTEASOME_DEGRADATION
KEGG_UBIQUITIN_MEDIATED_PROTEOLYSIS
BARHL1_TARGET_GENES
AAGWWRNYGGC_UNKNOWN
RAG1_TARGET_GENES
ZNF2_TARGET_GENES
NFMUE1_Q6
ZBTB12_TARGET_GENES
ZNF146_TARGET_GENES
NFE2L1_TARGET_GENES
E2F2_TARGET_GENES
SREBP1_Q6
ZNF596_TARGET_GENES
SKIL_TARGET_GENES
RCGCANGCGY_NRF1_Q6
KEGG_UBIQUITIN_MEDIATED_PROTEOLYSIS
GOBP_INSULIN_SECRETION
GOBP_REGULATION_OF_INSULIN_SECRETION
GOBP_REGULATION_OF_HORMONE_LEVELS
GOBP_PROTEIN_LOCALIZATION_TO_EXTRACELLULAR_REGION
GOBP_PEPTIDE_TRANSPORT
GOBP_REGULATION_OF_PEPTIDE_TRANSPORT
GOBP_HORMONE_TRANSPORT
GOBP_LIPID_HOMEOSTASIS
GOBP_REGULATION_OF_HORMONE_SECRETION
GOBP_ESTABLISHMENT_OF_PROTEIN_LOCALIZATION
GOBP_REGULATION_OF_PROTEIN_SECRETION
GOBP_AMIDE_TRANSPORT
GOBP_SIGNAL_RELEASE
GOBP_RESPONSE_TO_MONOSACCHARIDE
GOBP_REGULATION_OF_ESTABLISHMENT_OF_PROTEIN_LOCALIZATION
GOBP_CELL_PROLIFERATION_IN_BONE_MARROW
OBP_NEGATIVE_REGULATION_OF_DNA_BINDING_TRANSCRIPTION_FACTOR_ACTIVIT
GOBP_CHEMICAL_HOMEOSTASIS
GOBP_SECRETION

For Review Only

GOBP_POSITIVE_REGULATION_OF_PROTEIN_BINDING
WP_16P112_PROXIMAL_DELETION_SYNDROME
WP_16P112_DISTAL_DELETION_SYNDROME
Type 2 diabetes
Body mass index
Adult body size
Medication use (drugs used in diabetes)
Waist circumference
Triglyceride levels
Brain morphology (MOSTest)
Body fat distribution (arm fat ratio)
Body mass index in physically active individuals
Metabolic syndrome
Body mass index (joint analysis main effects and physical activity interaction)
Sleep duration (short sleep)
BMI (adjusted for smoking behaviour)
Body mass index (joint analysis main effects and smoking interaction)
Body mass index and type 2 diabetes (pairwise)
BMI in non-smokers
Blood glucose levels
Extremely high intelligence
Hip circumference
Fasting glucose
Obesity
Type 2 diabetes (adjusted for BMI)
Refractive error
Body size at age 10
Estimated glomerular filtration rate
Waist circumference adjusted for body mass index
HDL cholesterol levels
Hand grip strength
Fruit consumption
Fasting blood glucose (BMI interaction)
Apolipoprotein A1 levels
Type 2 diabetes or prostate cancer (pleiotropy)
Loneliness (MTAG)
BMI in smokers
Crohn's disease
Waist-hip index
A body shape index
Circulating leptin levels or type 2 diabetes
Alzheimer's disease or fasting glucose levels (pleiotropy)
Chronic obstructive pulmonary disease or resting heart rate (pleiotropy)

For Review Only

Pulse pressure
Regular attendance at a gym or sports club
Walking pace
Weight
Fasting blood glucose
General cognitive ability
Regular attendance at a religious group
Systolic blood pressure
Glycemic traits (pleiotropy)
Fasting insulin
Waist-to-hip ratio adjusted for BMI
Loneliness
Triglycerides
Body mass index in physically inactive individuals
Body mass index variance
Body fat distribution (leg fat ratio)
Body fat percentage and type 2 diabetes (pairwise)
Coronary artery disease
Liver enzyme levels (gamma-glutamyl transferase)
FEV1
Liver enzyme levels (alanine transaminase)
Hemoglobin A1c levels
Intelligence (MTAG)
Subcortical volume (min-P)
Global electrical heterogeneity phenotypes
Age at first birth
Acute insulin response
Body mass index (adult)
Two-hour glucose
High density lipoprotein cholesterol levels
Blood sugar levels
Body fat percentage
Processed meat consumption
Peak insulin response
C-reactive protein levels
Brain morphology (min-P)
Fasting plasma glucose
General factor of neuroticism
Systolic blood pressure x alcohol consumption interaction (2df test)
Medication use (agents acting on the renin-angiotensin system)
HDL cholesterol
Myocardial infarction
Lung function (FVC)

For Review Only

Hypertriglyceridemia
Insulin-related traits (multivariate analysis)
Peak expiratory flow
Waist circumference variance
Triglyceride levels x long total sleep time interaction (2df test)
Body fat distribution (trunk fat ratio)
Alcohol use disorder (total score)
Schizophrenia
Alanine aminotransferase levels
Menarche (age at onset)
Medication use (HMG CoA reductase inhibitors)
Household income (MTAG)
Obesity (extreme)
Glycated hemoglobin levels
Hip circumference adjusted for BMI
Cortical surface area (MOSTest)
Inflammatory bowel disease
Body fat percentage and fasting glucose (pairwise)
Type 2 diabetes (time to event)
Diabetes (gestational)
Non-oily fish consumption
Metabolically unhealthy in normal weight
Body mass index (SNP x SNP interaction)
Homeostasis model assessment of beta-cell function
Chronic kidney disease
Multisite chronic pain
Lamb consumption
Breast cancer
Childhood body mass index
Fish- and plant-related diet
Blood urea nitrogen levels
Anxiety/tension (special factor of neuroticism)
Nonalcoholic fatty liver disease
Neuroticism
Fasting blood insulin adjusted for BMI
General risk tolerance (MTAG)
Coronary heart disease
Serum metabolite levels (CMS)
Educational attainment
Chloride levels
Fasting blood insulin
Systolic blood pressure x smoking status (ever vs never) interaction (2df test)
Youthful appearance (self-reported)

For Review Only

Height
Obese vs. thin
Chronic obstructive pulmonary disease or high blood pressure (pleiotropy)
Essential hypertension (time to event)
Cognitive ability
Glycemic traits
Body mass index and fasting glucose (pairwise)
Two-hour glucose challenge
Bipolar disorder or body mass index
Coffee consumption
Subcortical volume (MOSTest)
Age at first sexual intercourse
Systemic lupus erythematosus
Urinary sodium excretion
Morning person
Number of sexual partners
Total cholesterol levels
Heel bone mineral density
Medication use (diuretics)
Beef consumption
Depressive symptoms (binary sum-score)
Albumin-globulin ratio
Offspring birth weight
Systolic blood pressure x smoking status (current vs non-current) interaction (2df test)
Appendicular lean mass
Retinal vascular caliber
HDL cholesterol levels x alcohol consumption (regular vs non-regular drinkers) interaction (2df)
Hematological and biochemical traits
LDL cholesterol levels x alcohol consumption (regular vs non-regular drinkers) interaction (2df)
Creatinine levels
Pancreatic beta-cell glucose sensitivity
Childhood onset type 2 diabetes
Barrett's esophagus
Body composition traits
Primary tooth development (time to first tooth eruption)
Fasting blood insulin (BMI interaction)
Serum alkaline phosphatase levels
Chronotype
HDL cholesterol levels x alcohol consumption (drinkers vs non-drinkers) interaction (2df)
LDL cholesterol levels x alcohol consumption (drinkers vs non-drinkers) interaction (2df)
circulating leptin levels
Functional impairment in major depressive disorder
Adiposity

For Review Only

Lymphocyte count
Neutrophil percentage of white cells
Intelligence
Barrett's esophagus or Esophageal adenocarcinoma
Ventricular ectopy or supraventricular ectopy (pleiotropy)
Insulin secretion rate
Insulin levels
Age at adiposity rebound
Asthma and attention deficit hyperactivity disorder
C-reactive protein
Electrocardiogram morphology (amplitude at temporal datapoints)
HDL cholesterol levels in current drinkers
Allergic rhinitis
Alcohol use disorder (consumption score)
Mild age-related type 2 diabetes
Birth weight
Triglyceride levels in current drinkers
Waist-hip ratio
Diastolic blood pressure
Iron status biomarkers (ferritin levels)
Liver enzyme levels (alkaline phosphatase)
Fasting blood glucose adjusted for BMI
Proinsulin levels
Triglyceride levels x short total sleep time interaction (2df test)
Serum metabolite levels
Triglyceride levels x alcohol consumption (drinkers vs non-drinkers) interaction (2df)
Triglyceride levels x alcohol consumption (regular vs non-regular drinkers) interaction (2df)
Depressive symptoms (sum-score)
Incremental insulin
Ventricular ectopy
Osteoarthrosis (time to event)
Serum alpha1-antitrypsin levels
Estimated glomerular filtration rate in non-diabetics
Autism spectrum disorder or schizophrenia
Endometrial cancer (endometrioid histology)
Smoking initiation
Body mass index x sex x age interaction (4df test)
Cholesterol
Hip index
Cognitive ability
Urolithiasis
Chronic obstructive pulmonary disease or coronary artery disease (pleiotropy)
Hip circumference variance

For Review Only

N-glycan levels
Liver enzyme levels
Risk-taking tendency (4-domain principal component model)
Waist circumference and related phenotypes
Metabolic syndrome (multivariate analysis)
Mental health study participation (provided email address)
Psychosis proneness (hypomanic personality scale and perceptual aberration scale)
Glucosuria (moderate to severe)
Insulin disposition index
Insulin levels adjusted for BMI
Glucosuria
Breast size
Intraocular pressure
Basal cell carcinoma
Lung function (FEV1/FVC)
L-selectin levels
Severe insulin-deficient type 2 diabetes
Mild obesity-related type 2 diabetes
Raw vegetable consumption
Endometrial cancer
High light scatter reticulocyte count
Urate levels
Hemoglobin
Cognitive function
Nicotine dependence symptom count
Glucose homeostasis traits
Non-lobar intracerebral hemorrhage (MTAG)
Cardiovascular disease risk factors
Lipid traits (pleiotropy) (HIPO component 1)
Apolipoprotein B levels
Hematocrit
Basal metabolic rate variance
Basal metabolic rate
Triglycerides-Blood Pressure (TG-BP)
Metabolic traits
Body mass index (age <50)
Serum uric acid levels
Obesity (early onset extreme)
Non-melanoma skin cancer
Gut microbiota (beta diversity)
Intracranial aneurysm
Free thyroxine concentration
Alcohol consumption

For Review Only

Gastroesophageal reflux disease
Serum total protein level
Metabolic syndrome (bivariate traits)
Corrected insulin response
Remission after SSRI treatment in MDD or neuroticism
Corrected insulin response adjusted for insulin sensitivity index
Waist Circumference - Triglycerides (WC-TG)
Cholesterol efflux capacity
Birth length
Problematic alcohol use
Blond vs. brown/black hair color
Hyperuricemia
Cognitive ability (MTAG)
chr11p11
chr8p23
chr2p23
chr16p11
chr3p21
chr4q24
chr3q22
chr17q23
chr17q21
BIOCARTA_PPARG_PATHWAY
GSE3982_MEMORY_CD4_TCELL_VS_TH2_UP
DACOSTA_UV_RESPONSE_VIA_ERCC3_DN
NIKOLSKY_BREAST_CANCER_17Q21_Q25_AMPLICON
DACOSTA_UV_RESPONSE_VIA_ERCC3_COMMON_DN

For Review Only

thways by MsigDB

N_genes	N_overlap	p
836	30	8.52E-07
316	16	5.75E-06
66	7	2.76E-05
30	5	4.42E-05
298	14	5.05E-05
129	9	5.93E-05
1082	32	2.03E-05
115	9	2.39E-05
918	28	4.07E-05
995	29	6.64E-05
233	12	7.30E-05
1087	30	0.000130686
937	27	0.000144677
1552	38	0.000193379
1258	32	0.000336151
240	11	0.000397445
507	17	0.000489598
1577	37	0.000537236
917	25	0.00058243
129	9	5.93E-05
192	15	4.75E-08
159	13	2.24E-07
520	23	5.45E-07
356	18	1.52E-06
253	15	1.66E-06
192	13	1.93E-06
312	16	4.90E-06
148	11	4.92E-06
245	14	5.68E-06
1585	43	5.85E-06
252	14	7.83E-06
336	16	1.24E-05
464	19	1.62E-05
188	11	4.65E-05
504	19	5.01E-05
7	3	7.90E-05
167	10	8.48E-05
958	28	8.52E-05
913	27	9.45E-05

For Review Only

83	7	0.000121573
66	7	2.76E-05
30	5	4.42E-05
541	93	1.05E-78
1052	75	1.31E-34
484	48	4.39E-28
34	18	1.78E-25
159	28	1.73E-23
534	45	2.23E-23
1049	60	2.30E-22
104	22	1.65E-20
62	18	1.07E-19
104	21	3.53E-19
71	18	1.62E-18
78	18	1.02E-17
64	16	1.73E-16
65	16	2.27E-16
11	9	6.26E-16
47	14	9.13E-16
16	10	1.12E-15
72	16	1.33E-15
119	19	1.82E-15
123	19	3.43E-15
48	13	3.98E-14
48	13	3.98E-14
1391	56	4.97E-14
253	24	5.62E-14
462	31	1.39E-13
638	36	2.39E-13
510	32	3.40E-13
127	17	1.13E-12
112	16	1.91E-12
29	10	2.40E-12
367	26	4.34E-12
33	10	1.06E-11
60	12	1.95E-11
17	8	1.97E-11
528	30	2.37E-11
531	30	2.73E-11
370	25	3.19E-11
12	7	5.19E-11
39	10	6.79E-11
53	11	8.94E-11

For Review Only

564	30	1.19E-10
30	9	1.30E-10
70	12	1.32E-10
152	16	2.14E-10
44	10	2.50E-10
163	16	6.08E-10
63	11	6.42E-10
683	32	6.74E-10
16	7	7.17E-10
65	11	9.12E-10
733	33	9.76E-10
84	12	1.19E-09
149	15	1.49E-09
19	7	3.05E-09
21	7	6.88E-09
167	15	7.22E-09
7	5	8.36E-09
490	25	1.07E-08
173	15	1.17E-08
149	14	1.30E-08
134	13	2.95E-08
38	8	3.12E-08
214	16	3.12E-08
308	19	3.54E-08
16	6	3.81E-08
72	10	3.89E-08
9	5	4.91E-08
17	6	5.83E-08
17	6	5.83E-08
150	13	1.13E-07
19	6	1.25E-07
32	7	1.76E-07
32	7	1.76E-07
11	5	1.76E-07
85	10	1.95E-07
345	19	2.10E-07
48	8	2.14E-07
66	9	2.22E-07
67	9	2.54E-07
111	11	2.81E-07
196	14	4.04E-07
197	14	4.30E-07
143	12	4.98E-07

For Review Only

37	7	5.08E-07
24	6	5.86E-07
97	10	6.82E-07
14	5	7.39E-07
25	6	7.62E-07
179	13	8.74E-07
26	6	9.80E-07
627	25	1.16E-06
281	16	1.27E-06
186	13	1.35E-06
61	8	1.44E-06
131	11	1.49E-06
28	6	1.57E-06
133	11	1.73E-06
820	29	1.76E-06
401	19	2.02E-06
605	24	2.10E-06
3	3	2.35E-06
3	3	2.35E-06
3	3	2.35E-06
30	6	2.42E-06
9	4	3.70E-06
9	4	3.70E-06
9	4	3.70E-06
118	10	4.13E-06
50	7	4.25E-06
33	6	4.36E-06
177	12	4.74E-06
51	7	4.87E-06
180	12	5.63E-06
123	10	6.01E-06
10	4	6.11E-06
21	5	6.95E-06
129	10	9.18E-06
4	3	9.31E-06
224	13	1.04E-05
58	7	1.17E-05
58	7	1.17E-05
59	7	1.31E-05
24	5	1.41E-05
12	4	1.41E-05
85	8	1.79E-05
62	7	1.83E-05

For Review Only

1028	31	1.91E-05
13	4	2.02E-05
63	7	2.03E-05
26	5	2.13E-05
142	10	2.13E-05
5	3	2.30E-05
5	3	2.30E-05
5	3	2.30E-05
5	3	2.30E-05
88	8	2.31E-05
522	20	2.52E-05
178	11	2.82E-05
361	16	2.99E-05
28	5	3.11E-05
182	11	3.46E-05
94	8	3.73E-05
184	11	3.82E-05
674	23	3.84E-05
70	7	4.07E-05
6	3	4.56E-05
16	4	4.97E-05
50	6	5.18E-05
129	9	5.93E-05
101	8	6.25E-05
603	21	6.31E-05
17	4	6.43E-05
102	8	6.70E-05
33	5	7.12E-05
77	7	7.54E-05
104	8	7.70E-05
7	3	7.90E-05
7	3	7.90E-05
7	3	7.90E-05
7	3	7.90E-05
18	4	8.18E-05
18	4	8.18E-05
312	14	8.25E-05
481	18	8.70E-05
107	8	9.41E-05
80	7	9.62E-05
8	3	0.00012522
8	3	0.00012522
8	3	0.00012522

For Review Only

408	16	0.000126285
286	13	0.000130095
38	5	0.00014256
21	4	0.000155093
2	2	0.000177454
2	2	0.000177454
2	2	0.000177454
2	2	0.000177454
9	3	0.000185978
22	4	0.000187579
380	15	0.000191021
90	7	0.000202156
120	8	0.000209152
24	4	0.000266829
24	4	0.000266829
230	11	0.000276709
68	6	0.000291838
96	7	0.000301414
536	18	0.000329339
46	5	0.000356951
237	11	0.000357306
11	3	0.000358152
11	3	0.000358152
26	4	0.000367625
323	13	0.00042326
73	6	0.000429421
74	6	0.000462164
12	3	0.000472836
3	2	0.000527664
3	2	0.000527664
3	2	0.000527664
3	2	0.000527664
76	6	0.000533466
469	16	0.000594041
30	4	0.000646277
109	7	0.000650967
80	6	0.000701526
14	3	0.000767019
222	10	0.000828416
32	4	0.000830487
15	3	0.000949356
15	3	0.000949356
15	3	0.000949356

For Review Only

15	3	0.000949356
15	3	0.000949356
86	6	0.001027175
4	2	0.001046021
4	2	0.001046021
4	2	0.001046021
4	2	0.001046021
4	2	0.001046021
4	2	0.001046021
4	2	0.001046021
4	2	0.001046021
58	5	0.001047967
313	12	0.001066954
87	6	0.001091148
155	8	0.001149416
16	3	0.001156967
16	3	0.001156967
16	3	0.001156967
16	3	0.001156967
35	4	0.001171963
409	14	0.001253855
236	10	0.001315435
326	12	0.001507326
63	5	0.001523146
38	4	0.001601319
64	5	0.001634517
18	3	0.00165296
18	3	0.00165296
18	3	0.00165296
203	9	0.001667923
330	12	0.001669681
5	2	0.00172801
5	2	0.00172801
5	2	0.00172801
39	4	0.00176584
39	4	0.00176584
131	7	0.001905022
19	3	0.001943658
19	3	0.001943658
19	3	0.001943658
67	5	0.00200455
20	3	0.002264266
69	5	0.002283009

For Review Only

70	5	0.002432323
43	4	0.002541491
6	2	0.002569207
6	2	0.002569207
6	2	0.002569207
6	2	0.002569207
6	2	0.002569207
6	2	0.002569207
6	2	0.002569207
21	3	0.002615795
139	7	0.002663126
22	3	0.002999197
45	4	0.003005289
64	11	7.66E-10
57	10	3.73E-09
92	11	3.99E-08
97	11	6.96E-08
198	14	4.57E-07
22	5	8.90E-06
56	7	9.22E-06
54	6	8.04E-05
282	12	0.000427879
8	3	0.00012522
180	13	9.31E-07
836	30	8.52E-07
316	16	5.75E-06
453	18	4.04E-05

For Review Only

FDR P
0.005530793
0.018686574
0.045840954
0.045840954
0.045840954
0.045840954
0.013343642
0.013343642
0.015133788
0.016283263
0.016283263
0.023044926
0.023044926
0.026952196
0.041645413
0.044315096
0.049627418
0.049918152
0.04995457
0.011037434
0.000368011
0.000867971
0.001407562
0.002486854
0.002486854
0.002486854
0.004535501
0.004535501
0.004535501
0.004535501
0.005520763
0.008015876
0.009687987
0.025737585
0.025912281
0.03666831
0.03666831
0.03666831
0.038549021

For Review Only

0.047115772
0.016182139
0.016182139
4.65E-75
2.90E-31
6.48E-25
1.97E-22
1.53E-20
1.65E-20
1.45E-19
9.13E-18
5.26E-17
1.56E-16
6.51E-16
3.76E-15
5.89E-14
7.17E-14
1.85E-13
2.53E-13
2.91E-13
3.26E-13
4.24E-13
7.59E-13
8.01E-12
8.01E-12
9.56E-12
1.04E-11
2.46E-11
4.07E-11
5.57E-11
1.79E-10
2.91E-10
3.55E-10
6.19E-10
1.47E-09
2.57E-09
2.57E-09
3.00E-09
3.35E-09
3.82E-09
6.05E-09
7.70E-09
9.89E-09

For Review Only

1.29E-08
1.36E-08
1.36E-08
2.15E-08
2.46E-08
5.85E-08
6.05E-08
6.21E-08
6.47E-08
8.07E-08
8.47E-08
1.01E-07
1.24E-07
2.50E-07
5.54E-07
5.71E-07
6.49E-07
8.18E-07
8.77E-07
9.58E-07
2.14E-06
2.19E-06
2.19E-06
2.45E-06
2.60E-06
2.61E-06
3.24E-06
3.74E-06
3.74E-06
7.15E-06
7.79E-06
1.05E-05
1.05E-05
1.05E-05
1.15E-05
1.22E-05
1.23E-05
1.26E-05
1.42E-05
1.55E-05
2.21E-05
2.32E-05
2.65E-05

For Review Only

2.67E-05
3.05E-05
3.51E-05
3.76E-05
3.83E-05
4.35E-05
4.82E-05
5.63E-05
6.10E-05
6.42E-05
6.78E-05
6.94E-05
7.23E-05
7.90E-05
7.95E-05
9.03E-05
9.27E-05
0.000100954
0.000100954
0.000100954
0.000102848
0.000153198
0.000153198
0.000153198
0.00016942
0.000172516
0.000175397
0.000188887
0.000192409
0.000220576
0.000233096
0.000235076
0.000265302
0.000347178
0.000348996
0.000387006
0.000427224
0.000427224
0.000475342
0.000503173
0.000503173
0.000632712
0.000641314

For Review Only

0.00066408
0.000696718
0.000696762
0.000720002
0.000720002
0.000750479
0.000750479
0.000750479
0.000750479
0.000750479
0.000814272
0.000902696
0.000950604
0.000983647
0.001084298
0.00116212
0.001179429
0.001179429
0.001240759
0.00138259
0.001496255
0.001547295
0.001762309
0.001843612
0.001850206
0.001872487
0.00193899
0.002045056
0.002151233
0.002183171
0.002186024
0.002186024
0.002186024
0.002186024
0.002235259
0.002235259
0.002240347
0.002347848
0.002523967
0.002565001
0.003278699
0.003278699
0.003278699

For Review Only

0.003287137
0.003366485
0.003667604
0.003966967
0.004436362
0.004436362
0.004436362
0.004436362
0.004623338
0.004637089
0.00469594
0.004942213
0.005085143
0.006416956
0.006416956
0.006618574
0.00694292
0.007132387
0.007751721
0.008254285
0.008254285
0.008254285
0.008254285
0.008428719
0.009654247
0.00974455
0.010434055
0.010620808
0.011616477
0.011616477
0.011616477
0.011616477
0.011686079
0.012948917
0.014018515
0.014051356
0.015069194
0.016396424
0.01758328
0.01758328
0.019630365
0.019630365
0.019630365

For Review Only

0.019630365
0.019630365
0.02070202
0.02070202
0.02070202
0.02070202
0.02070202
0.02070202
0.02070202
0.02070202
0.02070202
0.02070202
0.020983434
0.021364288
0.022162678
0.022162678
0.022162678
0.022162678
0.022162678
0.022353171
0.023812482
0.024875217
0.028382634
0.028558994
0.029898038
0.03034999
0.03034999
0.03034999
0.03034999
0.030404693
0.030404693
0.031083114
0.031083114
0.031083114
0.031507425
0.031507425
0.033854309
0.034129702
0.034129702
0.034129702
0.035059825
0.039446368
0.039616922

For Review Only

0.04204308
0.043063409
0.043063409
0.043063409
0.043063409
0.043063409
0.043063409
0.043063409
0.043063409
0.043678841
0.044302009
0.049620906
0.049620906
2.30E-07
5.60E-07
3.99E-06
5.22E-06
2.74E-05
0.000395036
0.000395036
0.003016846
0.014262639
0.036564347
0.00486139
0.002899515
0.009796426
0.045819377

For Review Only

Ta

Name	Coefficient
Pancreatic_Islets__H3K27ac	9.40E-07
Pancreatic_Islets__H3K4me3	1.03E-06
A08.186.211.464.Limbic.System	1.93E-08
Pancreatic_Islets__H3K4me1	2.68E-07
A08.186.211.730.885.287.500.Cerebral.Cortex	1.74E-08
A08.186.211.464.405.Hippocampus	1.65E-08
Pancreas	1.57E-08

For Review Only

ble S10. Enriched Cell Types in GWAS for CKMs

SE	P value
1.98E-07	1.05E-06
2.38E-07	7.61E-06
4.95E-09	4.67E-05
7.14E-08	8.81E-05
4.85E-09	0.000167105
4.59E-09	0.000167197
5.01E-09	0.000877008

For Review Only

FDR P
0.000512797
0.001860954
0.009578704
0.014367721
0.01142514
0.01142514
0.046481446

For Review Only

Category
Coding_UCSC.bedL2_0
Coding_UCSC.extend.500.bedL2_0
Conserved_LindbladToh.bedL2_0
Conserved_LindbladToh.extend.500.bedL2_0
CTCF_Hoffman.bedL2_0
DGF_ENCODE.extend.500.bedL2_0
DHS_Trynka.extend.500.bedL2_0
Enhancer_Hoffman.bedL2_0
Enhancer_Hoffman.extend.500.bedL2_0
FetalDHS_Trynka.bedL2_0
FetalDHS_Trynka.extend.500.bedL2_0
H3K27ac_Hnisz.bedL2_0
H3K27ac_Hnisz.extend.500.bedL2_0
H3K27ac_PGC2.bedL2_0
H3K27ac_PGC2.extend.500.bedL2_0
H3K4me1_peaks_Trynka.bedL2_0
H3K4me1_Trynka.bedL2_0
H3K4me1_Trynka.extend.500.bedL2_0
H3K4me3_peaks_Trynka.bedL2_0
H3K4me3_Trynka.bedL2_0
H3K4me3_Trynka.extend.500.bedL2_0
H3K9ac_peaks_Trynka.bedL2_0
H3K9ac_Trynka.bedL2_0
H3K9ac_Trynka.extend.500.bedL2_0
Intron_UCSC.bedL2_0
Intron_UCSC.extend.500.bedL2_0
Promoter_UCSC.bedL2_0
Promoter_UCSC.extend.500.bedL2_0
Repressed_Hoffman.bedL2_0
Repressed_Hoffman.extend.500.bedL2_0
SuperEnhancer_Hnisz.bedL2_0
SuperEnhancer_Hnisz.extend.500.bedL2_0
TFBS_ENCODE.bedL2_0
TFBS_ENCODE.extend.500.bedL2_0
Transcribed_Hoffman.bedL2_0
TSS_Hoffman.bedL2_0
TSS_Hoffman.extend.500.bedL2_0
UTR_3_UCSC.bedL2_0
UTR_3_UCSC.extend.500.bedL2_0
WeakEnhancer_Hoffman.bedL2_0
WeakEnhancer_Hoffman.extend.500.bedL2_0

For Review Only

Table S11. Heritability Enrichment Across Genomic Functional anc

Enrichment	Coefficient
7.115998693	1.65E-07
2.025038025	-7.18E-08
13.2294577	6.34E-07
2.076379119	1.97E-08
-2.180684797	-2.11E-07
1.204532707	-3.63E-08
1.523873	1.60E-08
3.764247513	1.14E-07
2.137484148	-6.96E-08
2.546321946	1.67E-08
2.132448678	4.18E-08
1.435163992	-4.81E-08
1.455065726	3.05E-08
1.87269508	-9.61E-09
1.743691031	2.13E-08
2.711536317	4.84E-08
1.659939226	-2.87E-08
1.538051041	5.46E-08
4.328912171	6.72E-08
2.46943742	-2.40E-08
1.731245327	-1.38E-08
5.751716816	1.37E-07
3.407997811	7.48E-08
2.112674855	-1.65E-08
1.152307109	2.12E-07
1.231081553	-2.15E-07
2.852214627	1.04E-07
2.340102484	-6.89E-08
0.531929886	-4.33E-10
0.736843137	1.98E-08
1.746542538	-4.09E-09
1.741285755	6.37E-09
2.272107231	3.24E-08
1.604366394	1.01E-08
1.549802983	5.49E-08
5.439819917	4.33E-08
4.396606629	9.32E-08
5.636142795	1.01E-07
3.27293976	1.57E-08
5.232184667	1.90E-07
2.061937356	-2.38E-08

For Review Only

d Regulatory Regions

Coefficient_std_error	Coefficient_z-score	Enrichment FDR P
1.23E-07	1.341798972	0.000428045
3.84E-08	-1.872236865	0.020876053
1.04E-07	6.08088247	5.28E-10
1.64E-08	1.197502528	6.63E-11
1.30E-07	-1.627760052	0.052476981
1.93E-08	-1.880971352	0.038687239
3.00E-08	0.532220272	0.001153319
8.90E-08	1.274934442	0.003607272
5.70E-08	-1.221305149	0.007812869
7.69E-08	0.217220559	0.052046674
2.97E-08	1.405931627	3.45E-06
6.32E-08	-0.760509988	4.40E-06
6.30E-08	0.48419853	1.33E-07
5.01E-08	-0.19176358	6.71E-05
4.55E-08	0.468526455	2.89E-06
4.04E-08	1.199371377	0.000421887
3.76E-08	-0.764146602	0.000115141
2.96E-08	1.841746306	2.00E-11
9.24E-08	0.727738916	0.011568917
4.99E-08	-0.479994507	0.000115141
2.98E-08	-0.464968623	0.000495461
9.79E-08	1.39929414	0.001322231
5.73E-08	1.305525018	1.33E-07
3.58E-08	-0.461816804	1.59E-07
1.47E-07	1.438463405	0.019461088
1.47E-07	-1.4691081	3.15E-06
1.58E-07	0.656491879	0.009072751
1.34E-07	-0.514193181	0.004006447
2.79E-08	-0.015534502	0.014854069
2.19E-08	0.902514988	4.54E-10
2.16E-07	-0.018934394	1.71E-07
2.13E-07	0.029864051	4.17E-08
4.90E-08	0.661396586	0.017282175
2.69E-08	0.376579714	0.001153319
2.47E-08	2.225439989	0.01022472
1.65E-07	0.262870206	0.007870434
1.12E-07	0.833200752	0.00013256
1.61E-07	0.625742882	0.011711036
8.68E-08	0.180692426	0.016192725
1.24E-07	1.533751277	0.026228254
4.28E-08	-0.556502629	0.018556614

For Review Only

Table S12. Polygenic Risk Score and Genetic Contribution Ac

Chromosome
chr1
chr2
chr3
chr4
chr5
chr6
chr7
chr8
chr9
chr10
chr11
chr12
chr13
chr14
chr15
chr16
chr17
chr18
chr19
chr20
chr21
chr22

Only

cross Chromosomal Regions

PRS Score Sum
-0.447429601
-0.284526036
-0.196584692
-0.508420795
-0.271107612
-0.154525857
-0.615275424
-0.447360414
-0.126785788
-0.193802023
-0.120198192
-0.539177329
-0.192191419
-0.214457284
-0.172925656
0.215703827
0.026504487
-0.210800671
-0.142466888
-0.145361558
0.011305517
-0.399001812

r Review Only

Table S13. Estimates of sample overlap and cryptic relatedness across the six

Traits	CAD	25(OH)D	BMI	CKD
**T2D**	0.037	-0.054	0.058	0.01
**CAD**	0.886	-0.01	0.009	0.014
**25(OH)D**	-0.01	1.052	-0.01	0.006
**BMI**	0.009	-0.01	0.793	0.017
**CKD**	0.014	0.006	0.017	1.019
**FG**	0.009	-0.005	0.038	0.006

For Review Only

source GWAS traits.

FG
0.025
0.009
-0.005
0.038
0.006
0.996

For Review Only
